# Alpha-enolase influences ATP pool of cytoplasm and lactate homeostasis by regulating glycolysis in gastric cancer

**DOI:** 10.1038/s41392-025-02451-0

**Published:** 2025-10-31

**Authors:** Xiong Shu, Shiya Liu, Ting Yang, Xuanyu Zhou, Gaigai Shen, Lixin Sun, Long Yu, Yuanting Cao, Yuliang Ran

**Affiliations:** 1 Beijing Research Institute of Orthopaedics and Traumatology, Beijing Jishuitan Hospital, Capital Medical University, Beijing, P. R. China; 2 State Key Laboratory of Molecular Oncology, National Cancer Center/National Clinical Research Center for Cancer/Cancer Hospital, Chinese Academy of Medical Sciences and Peking Union Medical College, Beijing, P. R. China; 3 Department of Epidemiology & Population Health, Stanford University of Medicine, Stanford, CA USA

**Keywords:** Cell biology, Gastrointestinal cancer

## Abstract

Glycolysis is crucial for maintaining cancer stemness. This study demonstrated the role of the glycolytic enzyme alpha-enolase (ENO1) in glycolysis and stemness in gastric cancer (GC). High ENO1 expression was associated with poor prognosis and promoted malignant phenotypes and stem-like characteristics in patients with GC. Mechanistically, ENO1 directly stimulates lactate and ATP production by regulating glycolysis, affecting lactate homeostasis and intracellular ATP pools, and coregulating the AMPK/mTOR and PI3K/AKT signaling pathways. This ultimately drives GC stemness, epithelial‒mesenchymal transition (EMT)-related marker expression, self-renewal, migration, and invasion. Notably, the increase in the intracellular ATP pool can directly activate the PI3K/AKT pathway in a concentration-dependent manner, thereby further stimulating glycolysis to form a positive feedback loop. The functional role of lactate depends on the simultaneous presence of glycolysis-derived ATP to synergistically activate the PI3K/AKT pathway. Lactate homeostasis can also promote tumor stemness by increasing overall plactylation levels. Furthermore, pharmacological studies revealed that metformin combined with copanlisib significantly inhibited tumors by blocking the energy metabolism pathways PI3K/AKT and AMPK/mTOR. Our findings are the first to reveal the multifaceted role of ENO1 in mediating intracellular signaling and metabolic regulation to enhance stemness in GC. By establishing cell models with varying metabolite concentrations, we identified differential regulation of the PI3K/AKT and AMPK/mTOR pathways through lactate homeostasis and intracellular ATP pools, further confirming the metabolic crosstalk mechanism. Rationally, targeting multiple nodes along the ENO1-ATP/lactate-AMPK/PI3K/AKT-mTOR axis may be effective for GC treatment, as indicated by the significant suppression of tumor growth by metformin (which inhibits ATP production) plus syrosingopine (which disrupts lactate homeostasis). In conclusion, the complex interplay between metabolism and tumor stemness offers novel therapeutic directions and potential treatment strategies for GC.

## Introduction

Gastric cancer (GC) remains the fourth leading cause of cancer-related mortality globally, with a relatively high incidence in East Asia.^1^ Despite some improvements, GC is still associated with dismal survival due to resistance, even in high-income countries, confirming that a focus on novel therapeutic targets and strategies is needed.^2^ With increasing interest in characterizing tumor metabolic profiles, increasing evidence suggests that metabolism, primarily glycolysis, is intricately intertwined with tumor stemness.^3^ Thus, targeting stemness and tumor heterogeneity may be prioritized in any attempt to overcome the inherent limitations of currently available therapies.^4^ Recent studies have indicated that tumor cells exhibit active glycolytic activity and are strongly dependent on glycolysis;^5^ the abnormal increase in glycolytic intermediates or products is considered a hallmark of enhanced stemness and chemotherapy resistance in cancer cells.^6^ Therefore, the investigation of specific targets for inhibiting tumor metabolism holds clinical significance.^7^

Active glycolysis typically represents the malignant phenotype of tumors and is typically accompanied by increased levels of glycolytic enzymes and metabolic substrates; however, the mechanism by which glycolysis affects tumors is unclear.^8^ The metabolic enzymes that initiate metabolic flexibility, including HK2, PKM2, and LDHA (glycolytic rate-limiting enzymes), are potential targets for inhibiting tumor stemness.^9–11^ Although α-enolase (ENO1), a nonrate-limiting enzyme in glycolysis, has been proven to accelerate glycolysis in cancer cells,^12^ few studies have extensively investigated its role in promoting glycolysis itself and further regulation to drive tumor progression. However, ENO1, a critical RNA-binding protein and multifunctional oncoprotein, has attracted special attention in various cancers.^12,13^ Previous studies have focused primarily on the moonlighting functions of ENO1 in tumor progression,^14^ such as its role in perturbing lipid metabolism,^12^ influencing YAP1-dependent arachidonic acid metabolism,^15^ HGFR signaling,^16^ and ferroptosis.^17^ Our previous work revisited the molecular function of ENO1 as a glycolytic enzyme and preliminarily demonstrated that ENO1 enhances GC stemness properties by increasing glycolytic capacity,^18^ suggesting that ENO1 might be an ideal therapeutic target for the blockade of glycolysis. However, the unclear role of glycolysis in tumor progression and the widespread presence of glycolysis in all cell types pose a formidable challenge in instigating antitumor activity without the risk of completely inhibiting glycolysis.^13^

The PI3K/AKT signaling pathway plays a pivotal role in regulating diverse cellular processes, including metabolism, proliferation, survival, and metastasis, and is frequently hyperactivated in cancers,^19,20^ including gastric cancer. In glycolysis-driven tumor progression and therapeutic resistance, this pathway serves as a metabolic hub that integrates intracellular energy availability with oncogenic signaling. Its activation in certain tumor subpopulations is essential for sustaining malignant behavior and cannot be bypassed by alternative pathways.^19,21^ While numerous inhibitors targeting key components of the PI3K/AKT pathway have demonstrated initial clinical efficacy, their long-term benefits are often limited by the emergence of drug resistance. This resistance largely stems from the heterogeneous and multifactorial mechanisms underlying pathway activation, including both genetic alterations and metabolic adaptations.^19,22^ These limitations underscore the need for combination strategies targeting both PI3K signaling and tumor metabolism.

On the basis of the above considerations, we further investigated the detailed mechanisms by which ENO1 regulates cancer cell stemness through glycolysis. We established GC cell line models with different concentrations of metabolites and found that ENO1 could regulate intracellular adenosine triphosphate (ATP) levels and lactate homeostasis. This, in turn, acts through specific mechanisms on downstream pathways, resulting in varying degrees of PI3K/AKT pathway activation and AMPK/mTOR pathway inactivation. Additionally, pharmacodynamic studies have demonstrated that combination therapy targeting multiple nodes in these pathways may alleviate the cancer burden.

## Results

### Upregulation of ENO1 in GC is associated with poor prognosis and stem-like traits

**Fig. 1:**
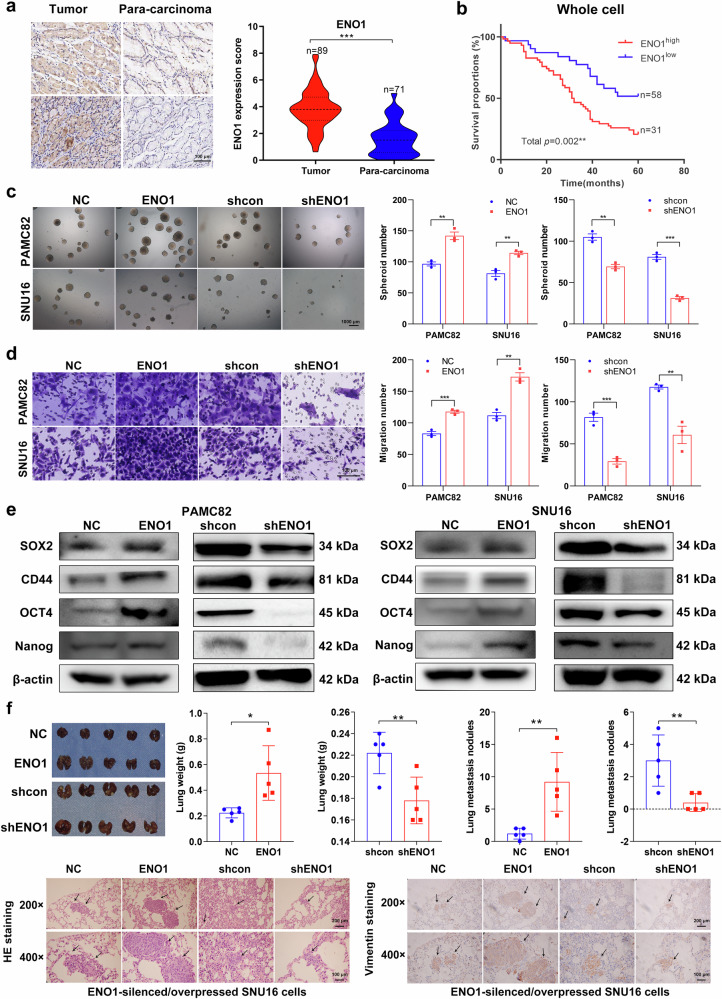
High expression of α-enolase (ENO1) is correlated with poor patient prognosis and with the stem-like traits of gastric cancer (GC). **a** ENO1 expression in normal tissues and GC tissues. Scale bar, 100 μm. **b** Analysis of the relationship between ENO1 protein expression and the survival rate of patients with GC. High and low ENO1 expression levels were defined on the basis of IHC immunoreactive score (IRS) analysis. The optimal cutoff value (IRS = 3.44) was determined via Youden’s J statistic, with samples classified as ENO1^high^ (IRS ≥ 3.44) or ENO1^low^ (IRS < 3.44) accordingly. Analysis of the sphere formation (**c**) and migration (**d**) abilities of PAMC82 and SNU16 cells stably expressing ENO1 compared with those of normal control (NC) cells. Scale bar, 1000 μm for sphere formation and 100 μm for migration. **e** The expression of stemness markers was analyzed by western blotting in PAMC82 and SNU16 cells stably expressing ENO1 and shENO1. **f** Results from the lung metastasis model of ENO1-silenced SNU16 cells. Scale bar, 100 μm. The data are presented as the means ± standard errors of the means (S.E.M.) of three independent experiments. *p < 0.05, **p < 0.01, ***p < 0.001

To investigate the expression of ENO1 in GC, we used a tissue microarray of human GC samples followed by immunohistochemistry (IHC) analysis. Compared with those in paracarcinoma normal tissues, ENO1 levels, both in the nucleus and cytoplasm, were significantly elevated in GC tissues (*p* < 0.001; Fig. 1a; Supplementary Fig. 1a). In addition, survival analysis of GC patients with balanced characteristics (Supplementary Fig. 1b) stratified by ENO1 expression revealed a significant decrease in the survival rate of patients with high ENO1 expression compared with those with low ENO1 expression (Fig. 1b). In addition, in patients with low nuclear ENO1 expression, survival is affected by the level of ENO1 expression in the cytoplasm (Supplementary Fig. 1c). ENO1 differentially expressed cell lines were successfully constructed from PAMC82 and SNU16 cell lines via stable lentivirus transfer and verified via western blotting (Supplementary Fig. 1d). We subsequently explored the functions of ENO1 in GC cell lines. Compared with the normal control (NC), the upregulated ENO1 had no significant effect on cell proliferation (Supplementary Fig. 1e) but promoted sphere formation (Fig. 1c), migration (Fig. 1d), invasion ability (Supplementary Fig. 1f), and stemness factor expression (Fig. 1e; Supplementary Fig. 1g), whereas the opposite trend in sphere formation, migration/invasion ability, and stemness factor expression was observed in ENO1-knockdown (shENO1) GC cells. In addition, we established a mouse model and reported that high ENO1 expression significantly increased lung metastasis and led to increased lung weight. Owing to the excessive number of metastatic nodules, impaired lung function may lead to systemic hypoxia, further affecting overall metabolism and physiological functions and ultimately causing a slight decrease in body weight in nude mice (Fig. 1f; Supplementary Fig. 1h). Taken together, these findings indicate that high ENO1 expression may be related to the poor prognosis of GC patients and the stem cell-like properties of GC cells.

### High ENO1 enhances GC stemness by mediating PI3K/AKT activation and AMPK/mTOR inactivation

**Fig. 2:**
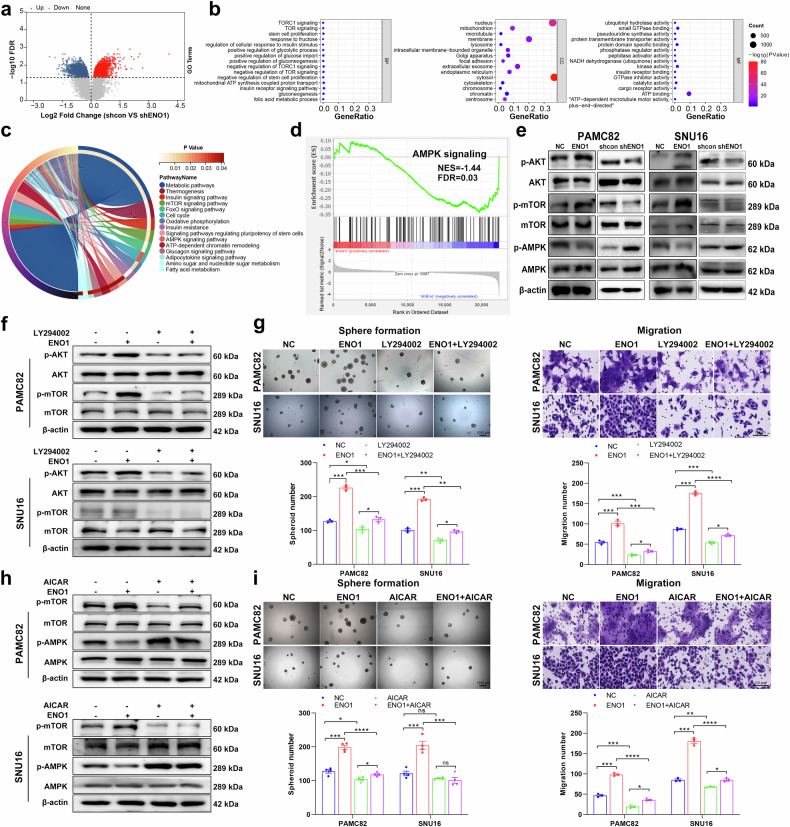
α-enolase (ENO1) promotes stem cell-like properties by regulating the PI3K/AKT and AMPK/mTOR pathways. **a** Volcano plots showing the differentially expressed genes (DEGs) in gastric cancer (GC) cells stably expressing shcon or shENO1. **b**, **c** Gene Ontology (GO) enrichment analysis of DEGs, with a significance threshold of a *p* value < 0.05. **d** Gene set enrichment analysis (GSEA) plot showing ENO1 expression in association with the AMPK/mTOR pathway. NES normalized enrichment score, FDR false discovery rate. **e** The expression of proteins related to the PI3K/AKT and AMPK/mTOR pathways was analyzed via western blotting in PAMC82 and SNU16 cells stably expressing ENO1 and shENO1. The expression of proteins related to the PI3K/AKT pathway was analyzed by western blotting (**f**), and sphere formation and migration (**g**) in PAMC82 and SNU16 cells treated with the PI3K inhibitor LY294002 (10 μM, 24 h) were analyzed. The expression of proteins related to the AMPK pathway was analyzed by western blotting (**h**), and sphere formation and migration (**i**) were analyzed in PAMC82 and SNU16 cells treated with the AMPK activator AICAR (0.5 mM, 24 h). Scale bar, 1000 μm for sphere formation and 100 μm for migration. The data are presented as the means ± SEMs of three independent experiments. *p < 0.05, **p < 0.01, ***p < 0.001, ****p < 0.0001, ns not significant

RNA-sequencing (RNA-Seq) analysis of cells in the control group (shcon) and ENO1-knockdown group (shENO1) was performed. In total, 2116 genes were upregulated and 1845 were downregulated in the ENO1-knockdown cells compared with the control cells (Fig. 2a). Kyoto Encyclopedia of Genes and Genomes (KEGG) and Gene Ontology (GO) analyses revealed that the differentially expressed genes (DEGs) were enriched mainly in metabolic pathways, such as positive regulation of glycolytic processes and AMPK/mTOR signaling (Fig. 2b, c). Gene set enrichment analysis (GSEA) plots further revealed that ENO1 may mediate the AMPK/mTOR pathway (Fig. 2d). Given the correlation between ENO1 and glycolysis-related processes indicated by bioinformatics analysis and the consideration that the PI3K/AKT pathway is a classical regulatory pathway of glycolysis,^23^ we hypothesized that ENO1 might also play a functional role in the PI3K/AKT pathway. Therefore, we performed western blotting analysis to validate these assumptions and observed that the differential expression of ENO1 significantly altered the phosphorylation status of the AKT/mTOR pathway and AMPK (Fig. 2e; Supplementary Fig. 2a).

We utilized the PI3K inhibitor LY294002 and the activator 740Y-P to verify whether ENO1 affects stemness via the PI3K/AKT pathway. As expected, the addition of LY294002 reversed the ENO1 overexpression-induced activation of the PI3K/AKT pathway (Fig. 2f; Supplementary Fig. 2b), as evidenced by reduced phosphorylation of AKT and mTOR; accordingly, the sphere formation, migration, and invasion abilities (Fig. 2g; Supplementary Fig. 2c) were significantly inhibited in the ENO1-overexpressing group after LY294002 treatment. In parallel, increased expression of p-AKT and p-mTOR was observed in shENO1 GC cells treated with 740Y-P, suggesting that 740Y-P treatment rescues the suppression of PI3K/AKT signaling caused by ENO1 deficiency (Supplementary Fig. 3a); in addition, 740Y-P reversed the inhibitory effects on self-renewal, migration, and invasion (Supplementary Fig. 3a) in shENO1 GC cells.

We also used the AMPK activator AICAR and the mTOR inhibitor rapamycin/activator MHY1485 to identify whether ENO1 can inactivate the AMPK/mTOR pathway and affect stem cell-like properties. AICAR increased the level of phosphorylated AMPK in ENO1-overexpressing cells, indicating reversion of AMPK/mTOR pathway inactivation induced by ENO1 (Fig. 2h; Supplementary Fig. 2d); AICAR also weakened the sphere formation, migration, and invasion abilities (Fig. 2i; Supplementary Fig. 2e) of ENO1-overexpressing GC cells. Moreover, the effects of rapamycin were similar to those of AICAR, with activation of the AMPK/mTOR pathway and inhibition of stem-like characteristics (Supplementary Fig. 3b), whereas the opposite trend was observed for MHY1485 compared with rapamycin (Supplementary Fig. 3c). Taken together, these findings suggest that ENO1 enhances stemness by activating PI3K/AKT signaling and inactivating the AMPK/mTOR pathway.

### Targeting both the PI3K/AKT pathway and the AMPK/mTOR pathway simultaneously has promising antitumor effects

On the basis of the aforementioned findings, we investigated whether ENO1 regulated stemness properties by synergetic mediation of the AMPK/mTOR and PI3K/AKT pathways in vitro and in vivo. Simultaneous treatment with AICAR plus LY294002 strongly inhibited the self-renewal (Supplementary Fig. 4a), migration (Supplementary Fig. 4b), and invasion abilities of ENO1-overexpressing GC cells (Supplementary Fig. 4c). We then wondered whether combined signaling pathway inhibition has potential clinical significance. Similar to the results from the in vitro models, the combination of metformin (AMPK activator; MET) and copanlisib (PI3K inhibitor; COPAN) significantly restrained cell viability (Supplementary Fig. 5a), colony formation (Supplementary Fig. 5b), sphere formation (Supplementary Fig. 5c), migration (Supplementary Fig. 5d), and invasion (Supplementary Fig. 5e) compared with those of the monotherapies. Finally, we constructed a mouse xenograft model via the subcutaneous injection of GC cells to verify the effects of MET and COPAN on proliferation in vivo (Supplementary Fig. 5f). The tumor size and weight in the combined MET and COPAN group were lower than those in the monotherapy group but not significantly different. Therefore, we aimed to identify a more effective combination treatment strategy. Taken together, these findings indicate that high ENO1 levels enhance stemness via synergistic mediation of PI3K/AKT activation and AMPK/mTOR inactivation, promoting GC growth.

### ENO1 can activate glycolysis to increase the production of lactate and cytoplasmic ATP and subsequently regulate the PI3K/AKT and AMPK/mTOR pathways, ultimately promoting tumor metastasis and stemness

Because the PI3K/AKT and AMPK/mTOR signaling pathways are classical pathways involved in energy metabolism, we next analyzed tumor samples from the TCGA database and found that patients with high expression of ENO1 (a key enzyme in glycolysis) presented higher glycolysis-related scores (Supplementary Fig. 6a). Combining the data shown in Supplementary Fig. 6b, our analysis of clinical gastric cancer samples from TCGA and RNA-Seq results consistently revealed that ENO1 influenced the levels of two key glycolytic products, ATP and lactate.

**Fig. 3:**
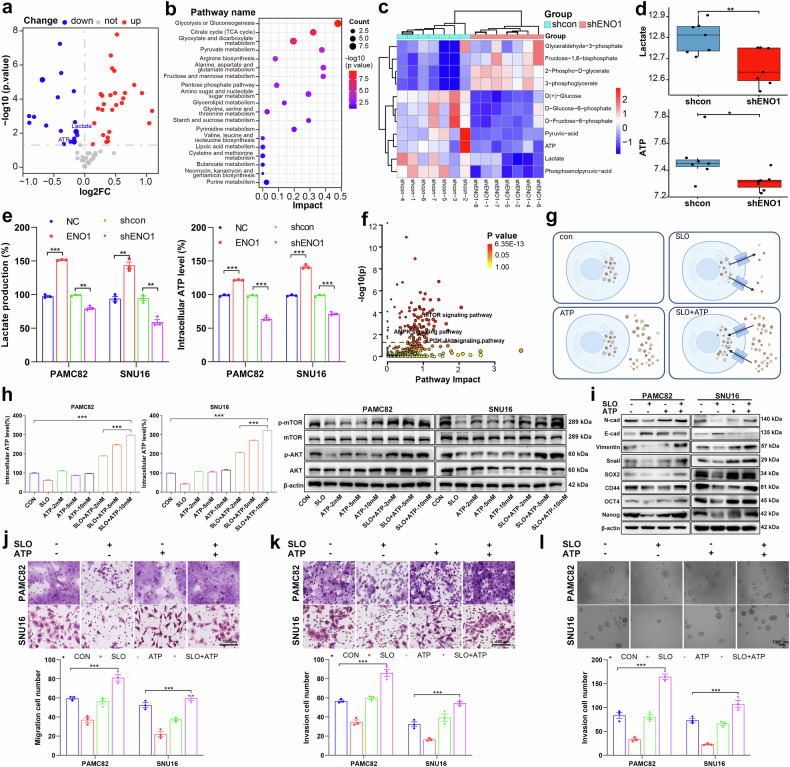
α-enolase (ENO1) promotes tumor invasion and stemness by modulating lactate and ATP production to activate the PI3K/AKT pathway. **a** Volcano plot of differentially expressed metabolites in shcon and shENO1 cells. **b** KEGG enrichment analysis of differentially expressed metabolites. **c** Heatmap of key intermediate glycolysis metabolites in different groups. **d** Box plots comparing ATP and lactate levels between the shcon and shENO1 groups. **e** Lactate and ATP production levels in PAMC82 and SNU16 cells were measured via a biochemical assay kit. **f** Integrated pathway analysis showing enrichment of differentially expressed genes (DEGs) and metabolites in the PI3K/AKT and AMPK signaling pathways. **g** Schematic of the membrane permeabilization assay used to establish cells with varying intracellular ATP concentrations. **h** Western blot showing PI3K/AKT signaling activation at different intracellular ATP concentrations postpermeabilization. **i** Western blot analysis of stemness and epithelial-mesenchymal transition (EMT) marker expression after ATP addition and membrane recovery. **j**–**l** Functional assays showing migration (**j**), invasion (**k**), and sphere formation (**l**) abilities after ATP addition and membrane recovery. The membrane permeability was induced by streptolysin O (SLO). Scale bar, 1000 μm for sphere formation and 100 μm for migration/invasion.The data are presented as the means ± SEMs of three independent experiments. *p < 0.05, **p < 0.01, ***p < 0.001

Next, energy metabolism profiling was performed on cells in the shcon and shENO1 groups (Supplementary Fig. 6c). Compared with those in the shcon group, 21 metabolites were upregulated, and 13 metabolites were downregulated in the shENO1 group (Fig. 3a). Next, KEGG enrichment analysis revealed that the differentially expressed metabolites were enriched mainly in the glycolysis and gluconeogenesis pathways, particularly in the positive regulation of glycolysis (Fig. 3b). The key intermediate metabolites of glycolysis exhibited significant differences, with ATP and lactate production markedly reduced (Figs. 3c, d). We further confirmed that ENO1 contributed to increased levels of lactate and ATP via the use of a biochemical assay kit to validate glycolytic products (Fig. 3e). Integrated pathway analysis of the DEGs and differentially expressed metabolites revealed significant enrichment of the PI3K/AKT and AMPK signaling pathways (Fig. 3f). To further verify whether ENO1-induced stem-like characteristics can be reversed, we treated cells with the glycolysis inhibitor 2-deoxy-D-glucose (2-DG) and examined the regulation of the PI3K/AKT and AMPK/mTOR pathways. Our results revealed that 2-DG decreased ATP and lactate levels, which was consistent with the findings in the shENO1 group (Supplementary Fig. 7a). The effects of ENOblock and 2-DG on the PI3K/AKT and AMPK/mTOR pathways were similar, suggesting that ENO1 primarily regulates these pathways via glycolysis (Supplementary Fig. 7b). Furthermore, 2-DG reversed the ENO1-induced increase in GC cell self-renewal, migration, and invasion capabilities (Supplementary Fig. 7c).

### An elevated cytoplasmic ATP pool directly activates PI3K/AKT signaling in a concentration-dependent manner, driving gastric cancer progression

On the basis of previously reported fundamental principles and bioinformatics findings, we inferred that ENO1 could regulate the PI3K/AKT and AMPK/mTOR pathways by activating glycolysis and increasing ATP and lactate levels, thereby influencing stemness.^24,25^ As shown in Supplementary Fig. 6d, ATP production was negatively correlated with the activation of the AMPK/mTOR pathway, which is consistent with previous reports in cancer cells.^26,27^ We subsequently validated the effects of ATP and lactate on the PI3K/AKT signaling pathway. Since ATP cannot freely diffuse across the plasma membrane, the intracellular ATP concentration is significantly greater than the extracellular ATP concentration. Therefore, we established cell populations with varying intracellular ATP concentrations through a membrane permeabilization assay and collected proteins 20 min post-treatment to investigate the transient effects of ATP on the PI3K/AKT pathway (Fig. 3g). The results demonstrated that under increased membrane permeability, ATP leakage due to ATP efflux could be partially restored by ATP supplementation, and PI3K/AKT signaling activation varied with the intracellular ATP concentration (Fig. 3h; Supplementary Fig. 8a). Additionally, flow cytometry analysis revealed that the membrane permeability induced by streptolysin O (SLO) recovered to approximately 80% after 8 h (Supplementary Fig. 8b). Thus, 12 h after ATP addition following membrane permeabilization, we allowed the membrane to recover before collecting cells to analyze its role in tumor stemness and epithelial‒mesenchymal transition (EMT; Fig. 3i; Supplementary Fig. 8c), as well as changes in functional phenotypes such as migration, invasion, and sphere formation (Figs. 3j–l). The changes in the expression of stemness-associated markers (downregulation of E-cadherin and upregulation of N-cadherin, vimentin, snail, SOX2, CD44, OCT4, and Nanog) indicated that high intracellular ATP levels could promote GC cell stemness by activating the PI3K/AKT signaling pathway. In addition, the results also revealed that after ATP addition and 12 h of recovery, the malignant phenotypes associated with migration, invasion, and stemness were significantly enhanced.

### Increased intracellular lactate promotes gastric cancer invasion and stemness in a concentration-dependent manner, which is mediated by glycolysis-derived ATP-mediated activation of the PI3K/AKT pathway

**Fig. 4:**
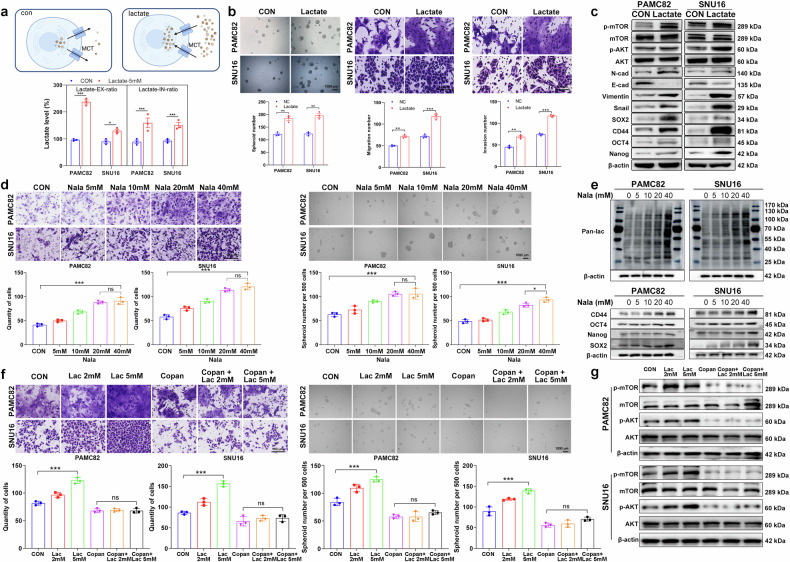
Lactate promotes tumor invasion and stemness in a dose-dependent manner, which is mediated by glycolysis-derived ATP-dependent PI3K/AKT activation. **a** Schematic and experimental results showing increased intracellular lactate levels after exogenous lactate supplementation (monocarboxylate transporter [MCT]). **b** Increased sphere formation, migration, and invasion abilities following lactate treatment. **c** Western blot analysis of stemness- and epithelial-mesenchymal transition (EMT)-related protein expression after lactate treatment. **d** Dose-dependent effects of sodium lactate on cell migration and sphere formation ability. **e** Western blot analysis of lactylation levels and stemness-related proteins after treatment with different sodium lactate concentrations. **f** Cell migration, sphere-forming ability, and PI3K/AKT pathway-related protein expression (**g**) under lactate treatment with or without the PI3K inhibitor copanlisib (Copan). Scale bar, 1000 μm for sphere formation and 100 μm for migration/invasion. The data are presented as the means ± SEMs of three independent experiments. ***p < 0.001, ns not significant

Since ENO1 can regulate lactate production, to further investigate the mechanism of lactate function, we found that exogenous lactate supplementation led to an increase in intracellular lactate levels, establishing a dynamic equilibrium between intracellular and extracellular lactate and promoting GC cell migration, invasion, and self-renewal (Figs. 4a, b). Western blotting analysis suggested that lactate enhanced stemness and EMT-related molecular phenotypes, thereby facilitating GC stemness and EMT (Fig. 4c; Supplementary Fig. 9a). Moreover, by adding different concentrations of sodium lactate, we observed a progressive increase in global lactylation levels (Fig. 4e), along with a gradual increase in molecular indicators of migration capacity (Fig. 4d), sphere formation ability (Fig. 4d), and stemness (Fig. 4e; Supplementary Fig. 9b). These findings suggested that lactate promoted tumor cell stemness by modulating overall lactylation levels. Building on the above discussion of downstream signaling pathways, we further explored the effect of lactate on the PI3K/AKT pathway and observed that lactate facilitated PI3K/AKT pathway activation. When lactate was coadministered with the PI3K inhibitor COPAN, its associated enhancement of migration and sphere formation (Fig. 4f) and activation of the PI3K/AKT pathway (Fig. 4g; Supplementary Fig. 9c) were significantly attenuated, indicating that the protumorigenic function of lactate was dependent on PI3K/AKT activation. Previously, we demonstrated that ATP activated the PI3K/AKT pathway in a concentration-dependent manner. Since ATP and lactate are both key glycolytic products, we further investigated their mechanisms of action by selectively inhibiting glycolysis and mitochondrial ATP production via 2-DG and oligomycin A (oligoA), respectively. Our results revealed that after 2-DG treatment, exogenous lactate did not enhance migration or sphere formation (Supplementary Fig. 9d) or activate the PI3K/AKT pathway (Supplementary Fig. 9e). In contrast, after oligoA treatment, exogenous lactate promoted migration and sphere formation (Supplementary Fig. 9f) and activated the PI3K/AKT pathway (Supplementary Fig. 9g). These findings suggest that the protumorigenic effects of lactate depend on glycolysis rather than mitochondria-derived ATP.

### Combined targeting of the ATP pool and lactate homeostasis with metformin and syrosingopine synergistically suppresses the stemness of GC cells, leading to more effective inhibition of tumor growth

**Fig. 5:**
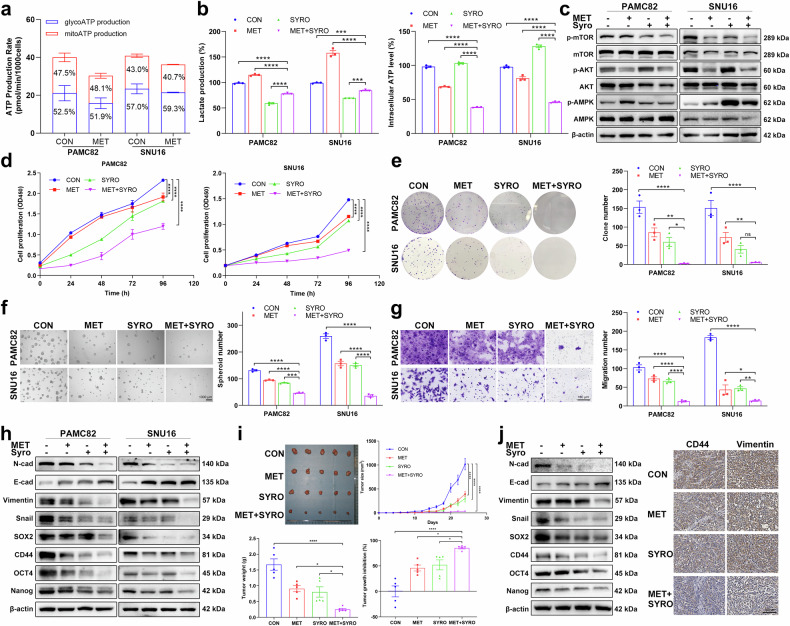
Simultaneous targeting of ATP and lactate inhibited the stemness of gastric cancer (GC) cells and tumor growth. **a** ATP production from glycolysis and mitochondria after metformin treatment. **b** ATP and extracellular lactate were tested in PAMC82 and SNU16 cells treated with metformin (MET; 10 mM, 48 h) and/or syrosingopine (Syro; 20 mM, 48 h). **c** Protein expression related to the PI3K/AKT and AMPK/mTOR pathways was tested in PAMC82 and SNU16 cells treated with metformin and/or syrosingopine. The synergistic inhibition of cell proliferation (**d**), colony formation (**e**), sphere formation (**f**), and migration (**g**) in PAMC82 and SNU16 cells treated with metformin and/or syrosingopine. Scale bar, 1000 μm for sphere formation and 100 μm for migration. **h** The expression of stemness markers in PAMC82 and SNU16 cells treated with metformin and/or syrosingopine was detected by western blotting. **i** Representative macroscopic tumor images obtained upon necropsy of mice postimplanted with MGC803 cells and postimplantation. Tumor volumes and weights were measured at the indicated time points in tumor-implanted mice after treatment with metformin and/or syrosingopine. **j** The protein expression and immunohistochemical images of stemness markers were tested in tumor samples collected from BALB/c nude mice treated with metformin (350 mg/kg, intraperitoneally [i.p.]) and/or syrosingopine (7.5 mg/kg, i.p.). Scale bar, 100 μm. The data are presented as the means ± standard errors of the means (S.E.M.) of three independent experiments. *p < 0.05, **p < 0.01, ***p < 0.001, ****p < 0.0001, ns not significant

Considering the synergy of cytoplasmic ATP and lactate, we propose a combination therapy that targets both metabolites. However, the direct inhibition of these two products in glycolysis by 2-DG may lead to significant toxicity, resistance to the drug, and tumor immunosuppression.^28^ Given the future clinical application value of this combination, we selected two clinically relevant drugs: MET and SYRO (SYRO). Various concentrations of MET affect the intracellular ATP pool, and a decrease in the intracellular ATP level but no change in the lactate level was detected (Supplementary Fig. 10a). Interestingly, our data also suggested a novel mechanism whereby MET can regulate ATP production primarily through glycolysis rather than through mitochondrial oxidative phosphorylation (Fig. 5a). In addition, extracellular lactate decreased after SYRO targeted monocarboxylate transporters (MCTs) to disrupt lactate homeostasis in tumors; in addition, an unexplained increase in ATP was also observed (Supplementary Fig. 10b), but this increase did not affect the inhibitory effects of either drug on the PI3K/AKT pathway or the activation of the AMPK pathway (Supplementary Fig. 10c, d). On the basis of the results of the metabolic product concentration and effects on both pathways from the dose gradient experiments, we used MET and SYRO for further investigations of synergistic effects on pathways and stemness. The inhibitory effect of the combination of MET and SYRO on the levels of intracellular ATP and lactate was verified (Fig. 5b). Significantly decreased expression of p-mTOR and p-AKT and increased p-AMPK were observed in the combination group, while total mTOR, AKT, and AMPK did not change (Fig. 5c; Supplementary Fig. 10e), suggesting a synergetic trend in AMPK/mTOR activation and PI3K/AKT inactivation, which was opposite to the trend in ENO1-overexpressing cells. As expected, the combination of MET and SYRO inhibited the proliferation (Fig. 5d), colony formation (Fig. 5e), self-renewal (Fig. 5f), migration (Fig. 5g), and invasion (Supplementary Fig. 10f) abilities of GC cells. In addition, this inhibition of the stemness of GC cells was also reflected in the significant downregulation of stemness and EMT markers (Fig. 5h; Supplementary Fig. 10g). In the subcutaneous xenograft model using MGC803 cells, the combined application of MET and SYRO resulted in the highest tumor suppression rate and the greatest reduction in tumor volume and weight (Fig. 5i). Decreased stemness was also observed in tumor tissues, as evidenced by western blotting and histological analysis of cells extracted from mice (Fig. 5j; Supplementary Fig. 10h).

**Fig. 6:**
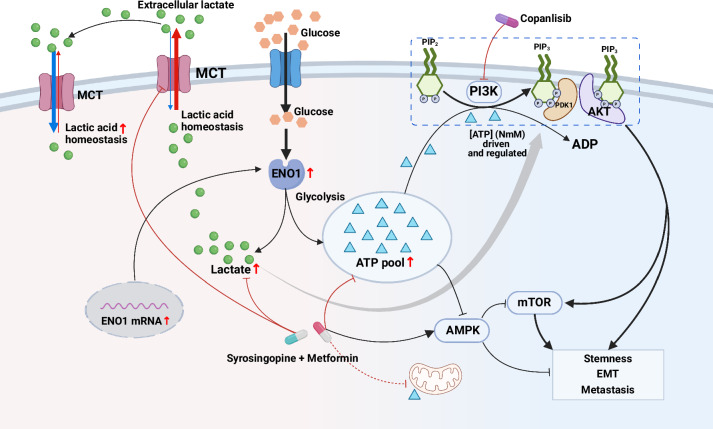
Schematic model of the role of α-enolase (ENO1) in regulating stemness in gastric cancer (GC). Mechanism by which ENO1 promotes gastric cancer stemness and progression by regulating glycolytic metabolites, modulating the ATP pool and lactate homeostasis, and synergistically activating the PI3K/AKT pathway

Overall, we concluded that ENO1 synergistically regulated the PI3K/AKT and AMPK/mTOR pathways through direct stimulation of glycolytic products and influenced the ATP pool and lactate homeostasis, ultimately promoting the stemness and tumor growth of GCs (Fig. 6).

## Discussion

Given that ENO1 is highly expressed on the plasma membrane of tumor cells, is detectable in circulation, and is functionally independent of upstream genetic alterations, it represents a promising and broadly applicable target for cancer therapy. These characteristics support its potential for both targeted treatments (such as antibody- or vaccine-based strategies)^13,25,29,30^ and noninvasive biomarker development.^31,32^ In addition to its established roles in interacting with YAP1, promoting MMP expression, and functioning as an RNA-binding protein,^15,17^ ENO1 has been increasingly recognized for its involvement in tumor progression. However, as a glycolytic enzyme, the role of ENO1 in regulating tumor progression and therapeutic resistance via glycolysis remains unclear. To our knowledge, this study is the first to reveal that ENO1 regulates the production and concentration of glycolytic metabolites in GCs, thereby sustaining malignant signaling within tumors. We propose novel insight into comprehensive cancer metabolism regulation: ENO1 enhances glycolysis, leading to the substantial production of ATP and lactate, which further elevates the intracellular ATP pool and lactate homeostasis. These metabolic changes, in turn, directly or indirectly modulate the AMPK/mTOR and PI3K/AKT/mTOR pathways, thereby promoting GC heterogeneity, stemness, and metastasis (Fig. 6).

Like the multifunctional roles of ENO1 in promoting cancer cell proliferation, invasion, and metastasis,^13^ we also observed these biological characteristics in GC cells overexpressing ENO1. In parallel, given the highly glycolytic phenotype of tumors (a hallmark of the Warburg effect) and the critical role of ENO1 in glycolysis,^13,33,34^ our further investigation revealed that ENO1 independently regulates glycolysis and significantly enhances the production of glycolytic metabolites such as ATP and lactate. Metabolomic data analysis and quantification of related metabolites demonstrated that ENO1 knockdown markedly suppressed lactate production and intracellular ATP levels in tumor cells. Moreover, treatment with 2-DG reversed the functional phenotypic changes induced by ENO1, further confirming that ENO1 regulated tumor stemness-related malignant phenotypes through glycolysis. Additionally, energy-responsive growth signaling pathways are frequently involved in metabolic reprogramming to support the aberrant proliferation and metastasis of tumor cells.^35–37^ Importantly, our data highlighted the dual enhancement of the energy-sensing AMPK/mTOR and PI3K/AKT/mTOR signaling pathways by ENO1-mediated glycolysis in GC, corroborating findings from previous studies.^25,38^ We validated their effects on tumor stemness-associated biological phenotypes by corresponding pathway inhibitors and activators and demonstrated that the oncogenic role of ENO1 via glycolysis is dependent on PI3K/AKT pathway activation. Additionally, considering the potential therapeutic targets within these pathways, we selected the clinically available MET (an AMPK activator) and COPAN (a PI3K inhibitor) to simultaneously inhibit both pathways (Figs. 2 and 3) and validated the feasibility of this combination therapy in both in vitro and in vivo models.

To further explore the metabolic regulatory mechanisms of ENO1, we conducted energy metabolomics analysis, which revealed that ENO1 knockdown led to a significant enrichment of differentially altered metabolites within the glycolytic pathway. Notably, the key glycolytic products ATP and lactate were markedly reduced. By integrating transcriptomic differentially expressed genes with metabolomic differentially altered metabolites for pathway analysis, we found significant enrichment of the previously identified PI3K-AKT and AMPK signaling pathways. Consequently, we propose an innovative mechanism whereby ENO1 increases the levels of cytoplasmic glycolytic intermediates (ATP and lactate) to cooperatively regulate these two energy-responsive growth signaling pathways, thereby sustaining tumor progression.

This study revealed that high levels of cytosolic ATP and lactate function not only as energy sources but also as signaling molecules for modulating metabolic reprogramming in cancer cells, cooperatively influencing the PI3K/AKT and AMPK signaling pathways to drive tumor progression.^25,39^ High concentrations of intracellular ATP promote tumor development not only through ATP-consuming processes and AMPK signaling but also by directly and concentration-dependently activating the PI3K/AKT pathway in an instantaneous manner; such activation facilitates EMT, tumor progression, and metastasis,^20,40^ with PI3K activation being ATP concentration dependent. Unlike previous studies suggesting that PI3K/AKT pathway activation enhances tumor glycolysis,^41^ we first propose that glycolysis itself could act as a driving force to produce ATP and subsequently activate PI3K/AKT, thereby influencing tumor progression. This finding is also consistent with prior research on T cells, which demonstrated that ATP may directly drive phosphatidylinositol 3,4,5-trisphosphate (PIP3) production, thereby establishing a positive feedback loop in PI3K/AKT signaling.^42,43^ Specifically, since ATP cannot freely diffuse across the cell membrane and is generated primarily through intracellular glycolysis and oxidative phosphorylation, the intracellular ATP pool varies among different tumor subpopulations. We established tumor cell models with varying cytoplasmic ATP concentrations through cell membrane permeabilization, which allowed ATP efflux. On the basis of the intracellular and extracellular ATP concentrations in tumor cells, exogenous ATP at different concentrations was subsequently supplemented to simulate the physiological environment. This approach partially restored ATP levels and concentration-dependently activated the PI3K/AKT pathway, indicating that the transient increase in intracellular ATP is a key factor in activating this pathway. This activation mechanism may involve ATP acting as a signaling molecule to facilitate the conversion of phosphatidylinositol 4,5-bisphosphate (PIP2) into PIP3, directly driving PIP3 production, which subsequently phosphorylates downstream AKT. Moreover, upon ATP supplementation following membrane permeabilization, GC cells presented a significant increase in malignant functional phenotypes and the expression of stemness-related markers. These findings suggest that ATP-mediated activation of the PI3K/AKT pathway triggers a cascade of oncogenic signaling events, promoting GC progression and metastasis. Traditionally, PI3K/AKT pathway activation is dependent primarily on tyrosine kinases (such as receptor tyrosine kinases (RTKs)), which increase PI3K enzymatic activity and catalyze the conversion of PIP2 to PIP3, thereby recruiting and activating AKT to initiate downstream signaling cascades that regulate cell proliferation, survival, and metabolism.^19^ However, our study demonstrated that activation of the PI3K/AKT pathway can be directly driven by ATP-mediated PIP3 production, independent of canonical tyrosine kinase signaling. This finding reveals a previously unrecognized mode of PI3K/AKT activation that is dependent on the intracellular ATP concentration rather than upstream receptor-mediated PI3K phosphorylation. This mechanism highlights a metabolic dimension of signal transduction, wherein ATP acts not only as an energy currency but also as a direct modulator of oncogenic signaling. Together with canonical kinase-based activation, this ATP-dependent mechanism supports a dual regulatory model of PI3K/AKT signaling, offering novel insights into hyperglycemia-induced resistance to PI3K inhibitors,^44^ and underscores the clinical potential of combined PI3K and glycolysis-targeted therapies.

In addition, extensive literature has demonstrated that PI3K/AKT pathway activation reprograms cellular metabolism by increasing the activity of the glucose transporter GLUT1 and metabolic enzymes,^20,40^ thereby increasing glucose uptake efficiency. This enhances glycolytic flux, generating more ATP and establishing a positive feedback loop that enables tumor cells to sustain high glycolytic activity, meeting the energy demands for rapid proliferation and growth while continuously reinforcing PI3K/AKT signaling to further drive tumor progression.

Additionally, lactate is transported across the cell membrane via MCTs.^45^ In this process, tumor cells with high glycolytic activity secrete substantial amounts of lactate into the extracellular space, where it is subsequently taken up by neighboring tumor cells with lower glycolytic activity.^46,47^ Consequently, lactate shuttling among heterogeneous tumor cell populations establishes stable lactate homeostasis, fostering a metabolic symbiotic relationship that promotes the malignant phenotype of the entire tumor cell community.^48^ In this study, we established tumor cell models with varying steady-state lactate concentrations via supplementation with extracellular lactate and found that lactate enhanced tumor cell stemness-related characteristics, such as migration and spheroid formation, by modulating global lactylation levels and activating the PI3K/AKT pathway. This observation is consistent with previous studies demonstrating that lactate can regulate the function of multiple proteins (e.g., MRE11 and NBS1) through lactylation, affecting protein activity and stability, influencing DNA repair and metabolic reprogramming, and ultimately contributing to the maintenance of tumor cell stemness.^49,50^ Furthermore, we conducted additional experiments in which 2-DG and oligoA were used to inhibit glycolysis-derived ATP and mitochondria-derived ATP, respectively. The results of our GC model demonstrated that the protumorigenic effects of lactate predominantly depend on glycolysis-derived ATP rather than mitochondria-derived ATP. This finding suggests that lactate does not function as a direct energy source through the TCA cycle but instead requires the simultaneous presence of glycolysis-derived ATP to exert its oncogenic effects, possibly linking to our proposed mechanism in which cytosolic ATP directly activates the PI3K/AKT pathway. Moreover, previous studies have identified AARS1/2 as ATP-dependent lactyltransferases that utilize ATP as an energy source to catalyze lactylation modifications through a two-step reaction.^51^ This evidence indirectly supports our hypothesis that the functional role of lactate is dependent on glycolysis-derived ATP, potentially through an ATP-coordinated lactylation modification mechanism. These findings provide a new perspective for further exploration of the cooperative mechanism between lactate and ATP in tumor progression.

Glycolytic metabolites exert dual regulatory effects on the activation of the PI3K/AKT/mTOR pathway in cancer cells. ATP, a key product of glycolysis, fuels the conversion of PIP2 to PIP3 by PI3K, thereby promoting downstream AKT phosphorylation. Moreover, lactate has been reported to increase PI3K phosphorylation,^52^ further contributing to pathway activation. Notably, ATP and lactate derived from glycolysis not only act as metabolic intermediates but also serve as signaling modulators in tumor cells with distinct metabolic and phenotypic heterogeneity. In particular, elevated cytosolic ATP levels in tumor subpopulations with high epithelial‒mesenchymal transition (EMT) states, along with lactate accumulation and uptake across the broader tumor cell population, influence both PI3K/AKT and AMPK signaling. These processes are tightly regulated by intracellular concentrations of cytosolic metabolites, where glycolysis- and OXPHOS-derived ATP, together with lactate import and export, maintain the ATP pool and lactate homeostasis. These findings also highlight additional therapeutic targets for modulating cytosolic ATP and lactate concentrations. Interestingly, MET not only reduces mitochondrial-derived ATP via the inhibition of complex I in the respiratory chain but also lowers glycolysis-derived ATP, as observed in PAMC82 and SNU16 cells. This dual effect on both ATP sources deviates from the traditional view that MET acts solely through mitochondrial pathways. MET can be used to target ATP synthesis in the cytoplasm, thereby affecting the intracellular ATP pool, whereas SYRO disrupts lactate transport and homeostasis by targeting MCTs. These two strategies represent effective therapeutic targets and potential combination treatment approaches for inhibiting tumor progression and metastasis.

Overall, this study is the first to reveal the potential interactions between ATP pool/lactate homeostasis and the PI3K-AKT/AMPK-mTOR signaling axis in GC, suggesting new directions for the development of anti-GC therapeutics. By investigating the glycolytic function of ENO1, we identified ENO1 as a specific metabolic target for GC and established a foundation for multitarget combination therapies involving the ENO1 signaling pathway, including those that target glycolysis, the two major tumor-promoting pathways, intracellular ATP pool regulation, and intracellular–extracellular lactate homeostasis. In terms of metabolic regulation, we innovatively proposed and demonstrated that an increased intracellular ATP pool can activate the PI3K/AKT pathway in a concentration-dependent manner, further stimulating glycolysis and establishing a positive feedback loop. Moreover, the functionality of lactate also depends on the concurrent presence of glycolysis-derived ATP to activate the PI3K/AKT pathway. Additionally, the microhomeostasis of lactate among different tumor cell subpopulations may promote tumor stemness by influencing global lactylation levels and related mechanisms. Although our study provides preliminary insights into the interactions among ENO1, glycolytic metabolism, and the AMPK/mTOR and PI3K/AKT signaling pathways, fully recapitulating the complexity of the human TME via only in vitro or in vivo models remains challenging. Consistent with previous reports highlighting the high toxicity of directly targeting glycolysis,^53^ our study demonstrated that cotargeting these two pathways yielded promising activity in vitro. However, the therapeutic efficacy in vivo has been limited due to drug-induced toxicity in mice. Notably, a striking synergistic therapeutic effect was achieved by simultaneously targeting intracellular ATP and lactate homeostasis. Further studies are needed to validate these findings and achieve a more comprehensive understanding of the interplay between metabolism and signaling pathways. In addition, to further elucidate the metabolic functions and mechanisms regulated by ENO1, future studies will incorporate real-time metabolic biosensors (e.g., for ATP, lactate, and pH) in combination with dynamic tracing approaches such as isotope-based flux analysis. Moreover, our discoveries regarding the relationship between metabolic processes and tumor therapy remain preliminary. ATP is a shared product of both glycolysis and oxidative phosphorylation, whereas lactate functions as a metabolic signal that transitions from glycolysis to oxidative phosphorylation, playing a pivotal role in the intricate metabolic landscape of tumors.^46,54^ The metabolic plasticity reflected in the interconversion between glycolysis and oxidative phosphorylation in GCs underscores the importance and feasibility of targeting both processes, providing further directions for exploration. The complexity of tumor metabolism highlights the necessity of elucidating these pathways and their potential compensatory mechanisms, which is crucial for a comprehensive understanding of their underlying processes.

In conclusion, our study revealed that ENO1, a glycolytic enzyme, stimulates the production of the glycolytic metabolites ATP and lactate by regulating glycolysis, thereby influencing the cytosolic ATP pool and overall lactate homeostasis in tumor cells. These processes collectively modulate the AMPK/mTOR and PI3K/AKT pathways, ultimately promoting stem-like characteristics and the progression of GC. In terms of metabolic regulation, we propose that the cytosolic ATP pool directly activates the PI3K/AKT pathway in a concentration-dependent manner. Increased intracellular lactate promotes gastric cancer invasion and stemness in a dose-dependent manner, which is mediated by glycolysis-derived ATP-dependent activation of the PI3K/AKT pathway. Additionally, lactate microhomeostasis can promote tumor stemness by affecting global lactylation levels. This study provides an in-depth analysis of the complex interplay between cytosolic metabolites and tumor stemness. Furthermore, our strategy of targeting multiple nodes along the ENO1-ATP/lactate-AMPK/PI3K/AKT-mTOR axis via clinically available drugs represents a novel translational approach that bridges laboratory research and clinical treatment for GC.

## Materials and methods

### Tissue microarray and IHC

The tissue microarray (consisting of 71 normal and 89 GC samples) was purchased from Shanghai Biochip Co. Ltd. (China). The study was approved by the medical ethics committee of the Cancer Hospital, Chinese Academy of Medical Sciences, Beijing, China (ethical approval number: NCC1999 G-003).

ENO1 expression in tissue samples was detected via a standard IHC procedure with a primary anti-ENO1 antibody (Abcam, USA; ab227978, 1:1000) and a horseradish peroxidase (HRP)-labeled goat anti-rabbit IgG secondary antibody (Jackson, USA). Then, the tissue microarrays were stained with 3,3’-diaminobenzidine solution (DAB; Agilent, USA) and counterstained with hematoxylin (Dako, USA). Finally, the slides were viewed via a bright field microscope (Olympus, USA). IHC staining was graded by blinded pathologists according to the average immunostaining intensity of ENO1. High and low ENO1 expression levels were defined on the basis of immunoreactive score (IRS) analysis. The optimal cutoff value (IRS = 3.44) was determined via Youden’s J statistic, with samples classified as ENO1^high^ (IRS ≥ 3.44) or ENO1^low^ (IRS < 3.44) accordingly.

### Cell lines and culture

Human GC cell lines, including PAMC82, SNU16, and MGC803, were obtained from the Chinese Academy of Sciences (China). The PAMC82 cell line was cultured in Dulbecco’s modified Eagle’s medium (DMEM; Invitrogen, USA), and the SNU16/MGC803 cell line was cultured in Roswell Park Memorial Institute-1640 medium (Gibco, USA), which was supplemented with 10% fetal bovine serum (FBS; Gibco, USA), 1% penicillin, and 1% streptomycin. The cell lines were maintained in a humidified atmosphere with 5% CO_2_ at 37 °C.

### Cell transfection for ENO1 silencing and overexpression

To silence ENO1, two cell lines were infected with lentiviruses containing short hairpin RNA (shRNA) targeting ENO1 (Obio Technology, China). After two days, cell lines stably expressing shENO1 (ENO1-knockdown) were enriched by a 2-day puromycin (Gibco, USA) treatment to select positive clones. To achieve stable overexpression of ENO1, cells were transfected with the pRLenti-CMV-ENO1-3FLAG-PGK-Puro plasmid (Obio Technology, China) via the Nucleofector Electroporation System (Lonza, Switzerland). Additionally, a blank pRLenti-CMV-MCS-3FLAG-PGK-Puro plasmid was transfected as a negative control.

### Western blotting

Western blotting was performed via a standard procedure as previously described.^25^ The primary antibodies against ENO1 (ab227978), CD44 (ab157107), SOX2 (ab97959), Nanog (ab80892), and OCT4 (ab18976) were from Abcam (USA), and those against AMPK (2532S), p-AMPK (2535S), AKT (2920S), p-AKT (4060S), mTOR (2983S), p-mTOR (5536S), β-actin (4970S), E-cad (3195S), N-cad (13116S), Vimentin (5741S), and Snail (3879S) were from Cell Signaling Technology, Inc. (USA). The secondary antibodies used were HRP-labeled goat anti-rabbit or anti-mouse IgG (Jackson, USA).

### Sphere formation assay

Self-renewal capacity was evaluated via a sphere formation assay. The cells (500 cells/well) were seeded in ultralow attachment 24-well plates (Corning, USA) as described previously^18^ and cultured at 37 °C for 7‒10 days. Then, the number of spheroids was determined via an inverted microscope (Nikon, Japan).

### Cell invasion and migration assays

To evaluate migratory and invasive activity, Transwell™ chambers (24-well insert; pore size, 8 μm; Corning, USA) were coated with or without diluted Matrigel (BD Biosciences, USA). A total of 5 × 10^4^ serum-starved cells were seeded in serum-free medium in the upper chamber. Complete medium containing 10% FBS was used as a chemoattractant in the lower compartment of the plate. After 24 h of incubation at 37 °C, noninvading cells in the upper chamber were removed. Invading cells were fixed with paraformaldehyde (Solarbio, China) and stained with 0.1% crystal violet (Solarbio, China). The infiltrating cells were counted in five random fields per well via an inverted microscope.

### RNA-Seq and bioinformatics analysis

The RNA-seq data were obtained from the TCGA Data Portal (https://tcga-data.nci.nih.gov/tcga/) and from cell samples. DEGs were defined as genes with an adjusted *p* value of <0.05. RNA-seq was used to identify the DEGs between shENO1 and CON (NC) GC cells to identify potential targets of ENO1 and was conducted as reported previously.^25^ Gene Ontology (GO) and Kyoto Encyclopedia of Genes and Genomes (KEGG) pathway enrichment analyses and gene set enrichment analysis (GSEA) were employed to characterize the ENO1 genes and DEGs to identify the potential pathways and networks involved in ENO1-mediated GC progression.

### Flow cytometry

PAMC82 and SNU16 cells (2 × 10⁵ cells per well in a 6-well plate) were collected, washed with precooled PBS, and fixed in 70% ethanol at 4 °C overnight. After fixation, the cells were washed and resuspended in 200 µL of PBS, followed by staining with PI dye (1:200) and incubation for 30 min at room temperature in the dark. Flow cytometry was conducted via a flow cytometer (Attune™ Nx, USA) according to the manufacturer’s instructions. Data analysis was performed via Flow Jo vX.0.7 software (BD Biosciences, USA). The kits used in this study were purchased from Beyotime Biotechnology (Shanghai, China).

### Colony formation assay

The cells treated with the indicated agents for 96 h were seeded into 6-well plates (500 cells/well) and cultured in complete growth medium in 5% CO_2_ at 37 °C for 2 weeks. Clones were then stained with 0.1% crystal violet (Solarbio, China) for 30 min at room temperature, followed by fixation with 4% paraformaldehyde (Solarbio, China) for 20 min. Colony numbers were counted under a light microscope (Nikon, Japan), and images were taken using a ChemiDoc MP Imaging System (Bio-Rad, USA).

### Cell proliferation assay

Cells at a density of 4000 cells/well were seeded in 96-well plates and incubated overnight at 37 °C in a humidified atmosphere with 5% CO₂. Then, the cells were treated with SYRO (SYRO; MedChemExpress, USA), COPAN (MedChemExpress, USA), MET (MET; Sigma, USA), or phosphate-buffered saline buffer for 72 h for cell proliferation analysis. Each compound was used at the indicated concentration. Cell proliferation was measured via a CCK-8 kit (Biosharp, China) according to the manufacturer’s instructions. The absorbance at 450 nm every 24 h was determined with a microplate reader for 96 h.

### Xenograft assays in BALB/c nude mice

All the animal studies were carried out in accordance with protocols approved by the ethics committee (number: NCC2020A049). BALB/c nude mice (4–5 weeks old) were obtained from HFK Bioscience Company (China). The mice were randomly grouped and housed in a controlled environment with a temperature of 22 ± 2 °C and a 12 h-light/dark cycle. For the tumorigenesis assay, 2.4 × 10^6^ MGC803 cells were subcutaneously inoculated into the backs of nude mice (5 mice/group). The mice were treated with MET (Sigma, USA), SYRO (MedChemExpress, USA), or vehicle control (Sigma, USA) via intraperitoneal injection every other day. COPAN (MedChemExpress, USA) was administered intraperitoneally twice per week for 3 weeks. Tumor size was measured every two days. After 25 days, the mice were euthanized, and the tumor xenografts from each group were harvested and weighed. In addition, cells in the tumor xenografts were subjected to IHC and western blotting analysis for stemness markers. For the mouse model of lung metastasis (established with ENO1-silenced/overexpressing SNU16 cells), 3 × 10^6^ cells/group (NC, ENO1, shCON, and shENO1) were injected via the tail vein into the mice. The mice were killed 7 weeks after injection, and the lungs were collected, weighed and used for IHC analysis.

### Supplementation and measurement of ATP and lactate

The cells were washed twice in buffer containing Mg^2+^, Ca^2+,^ and ATP-free Hanks’ balanced salt solution (HBSS) and then incubated for 20 min under the following conditions: (1) HBSS; (2) HBSS + 10 mM ATP; (3) HBSS + 200 U/ml streptolysin O (SLO); and (4) HBSS + 200 U/ml SLO + 10 mM ATP. Simultaneously, the cells were cultured with DMEM and 5 mM lactate for 48 h. After the corresponding treatments, the cells were collected, and ATP and lactate levels were measured.

Levels of lactate production were measured according to the instructions of the LAC Colorimetric Assay Kit II (Biovision, USA; K627-100). Lactic acid levels were determined with an iMark™ Microplate Absorbance Reader (Bio-Rad, USA) at an absorbance of 450 nm and normalized to that of the control (μmol/10^6^ cells). The ATP levels in the cell lysate were evaluated via a firefly luciferase-based ATP Assay Kit (Beyotime, China). Luminescence was measured via a VICTOR Nivo^TM^ Multimode Microplate Reader (PerkinElmer, USA) and was normalized to the protein concentration (nmol/mg). All the data were normalized to the control group (set as 100%).

### Targeted energy metabolomics profiling

To investigate the metabolic alterations induced by ENO1 knockdown, targeted energy metabolomics analysis was conducted via an LC‒MS/MS platform in both shENO1 and control (shcon) gastric cancer cells. All metabolites were detected and quantified by MetWare (http://www.metware.cn/) via an AB Sciex QTRAP 6500 + LC‒MS‒MS/MS system. The analytical system consisted of ultra-performance liquid chromatography (UPLC) (Waters ACQUITY H-Class, https://www.waters.com/nextgen/cn/zh.html) and tandem mass spectrometry (MS/MS) (QTRAP^®^ 6500 +, SCIEX, https://sciex.com.cn/). Metabolites were quantitatively analyzed via multiple reaction monitoring (MRM) in triple quadrupole mode. All metabolite concentrations were normalized on the basis of the protein content of each sample. The metabolomics data were analyzed via MetaboAnalyst 6.0 (https://www.metaboanalyst.ca/). Differentially abundant metabolites between the two groups were identified via a t test, and those with a VIP > 1 and a *p* value < 0.05 were considered statistically significant. These differentially abundant metabolites were subsequently subjected to KEGG pathway enrichment analysis to identify key metabolic pathways affected by ENO1 knockdown.

### Integrated pathway enrichment analysis

To comprehensively understand the biological pathways affected by ENO1 knockdown, integrated pathway analysis was performed via the Joint Pathway Analysis module in MetaboAnalyst 6.0 (https://www.metaboanalyst.ca/). The analysis was based on the combination of significantly altered metabolites and differentially expressed genes between the shENO1 and control groups. This approach allows the visualization of enriched KEGG pathways and the integration of gene–metabolite relationships within biological contexts. The resulting pathways were ranked on the basis of their statistical significance (*p* value) and pathway impact and visualized as a scatter plot.

### Statistical analysis

Data analyses and visualization were conducted via GraphPad Prism 5.0 (GraphPad Software, USA) and SPSS 13.0 (SPSS, Inc., USA). All the data are expressed as the means ± standard errors of the means (S.E.M.) or numbers (percentages, %) of at least three independent experiments. The relationships between ENO1 expression levels and clinicopathological parameters were determined via the chi-square test. Survival analysis was carried out via the Kaplan‒Meier method, with the log-rank test used for comparison. Statistical significance was calculated via Student’s t test or one-way analysis of variance as appropriate, and a *p* value < 0.05 was considered to indicate statistical significance.

## Supplementary information

Supplementary_Materials

## Data Availability

The raw sequencing datasets have been submitted to the Gene Expression Omnibus (GEO; https://www.ncbi.nlm.nih.gov/geo/query/acc.cgi?acc=GSE307717). All data supporting the findings of this study are available in the submitted material.

## References

[1] Thrift, A. P., Wenker, T. N. & El-Serag, H. B. Global burden of gastric cancer: epidemiological trends, risk factors, screening and prevention. *Nat. Rev. Clin. Oncol.* **20**, 338–349 (2023). 36959359 10.1038/s41571-023-00747-0

[2] Morgan, E. et al. The current and future incidence and mortality of gastric cancer in 185 countries, 2020-40: a population-based modeling study. *EClinicalMedicine* **47**, 101404 (2022). 35497064 10.1016/j.eclinm.2022.101404 PMC9046108

[3] Tufail, M., Jiang, C. H. & Li, N. Altered metabolism in cancer: insights into energy pathways and therapeutic targets. *Mol. Cancer* **23**, 203 (2024). 39294640 10.1186/s12943-024-02119-3 PMC11409553

[4] Philchenkov, A. & Dubrovska, A. Cancer stem cells as a therapeutic target: current clinical development and future prospective. *Stem Cells* **42**, 173–199 (2024). 38096483 10.1093/stmcls/sxad092

[5] Paul, S., Ghosh, S. & Kumar, S. Tumor glycolysis, an essential sweet tooth of tumor cells. *Semin Cancer Biol.* **86**, 1216–1230 (2022). 36330953 10.1016/j.semcancer.2022.09.007

[6] Chen, K. et al. The metabolic flexibility of quiescent CSC: implications for chemotherapy resistance. *Cell Death Dis.* **12**, 835 (2021). 34482364 10.1038/s41419-021-04116-6 PMC8418609

[7] Kroemer, G. & Pouyssegur, J. Tumor cell metabolism: cancer’s Achilles’ heel. *Cancer Cell* **13**, 472–482 (2008). 18538731 10.1016/j.ccr.2008.05.005

[8] Park, J. H., Pyun, W. Y. & Park, H. W. Cancer metabolism: phenotype, signaling and therapeutic targets. *Cells* **9**, 2308 (2020). 10.3390/cells9102308 PMC7602974 33081387

[9] De Francesco, E. M., Sotgia, F. & Lisanti, M. P. Cancer stem cells (CSCs): metabolic strategies for their identification and eradication. *Biochem. J.* **475**, 1611–1634 (2018). 29743249 10.1042/BCJ20170164 PMC5941316

[10] Zhao, H., Jiang, R., Feng, Z., Wang, X. & Zhang, C. Transcription factor LHX9 (LIM Homeobox 9) enhances pyruvate kinase PKM2 activity to induce glycolytic metabolic reprogramming in cancer stem cells, promoting gastric cancer progression. *J. Transl. Med.* **21**, 833 (2023). 37980488 10.1186/s12967-023-04658-7 PMC10657563

[11] Wu, C. et al. FOXQ1 promotes pancreatic cancer cell proliferation, tumor stemness, invasion and metastasis through regulation of LDHA-mediated aerobic glycolysis. *Cell Death Dis.* **14**, 699 (2023). 37875474 10.1038/s41419-023-06207-y PMC10598070

[12] Ma, Q. et al. The moonlighting function of glycolytic enzyme enolase-1 promotes choline phospholipid metabolism and tumor cell proliferation. *Proc. Natl Acad. Sci. USA* **120**, e2209435120 (2023). 37011206 10.1073/pnas.2209435120 PMC10104498

[13] Huang, C. K., Sun, Y., Lv, L. & Ping, Y. ENO1 and cancer. *Mol. Ther. Oncolytics* **24**, 288–298 (2022). 35434271 10.1016/j.omto.2021.12.026 PMC8987341

[14] Qiao, G., Wu, A., Chen, X., Tian, Y. & Lin, X. Enolase 1, a moonlighting protein, as a potential target for cancer treatment. *Int J. Biol. Sci.* **17**, 3981–3992 (2021). 34671213 10.7150/ijbs.63556 PMC8495383

[15] Sun, L. et al. ENO1 promotes liver carcinogenesis through YAP1-dependent arachidonic acid metabolism. *Nat. Chem. Biol.* **19**, 1492–1503 (2023). 37500770 10.1038/s41589-023-01391-6

[16] Li, H. J. et al. ENO1 promotes lung cancer metastasis via HGFR and WNT signaling-driven epithelial-to-mesenchymal transition. *Cancer Res.* **81**, 4094–4109 (2021). 34145039 10.1158/0008-5472.CAN-20-3543

[17] Zhang, T. et al. ENO1 suppresses cancer cell ferroptosis by degrading the mRNA of iron regulatory protein 1. *Nat. Cancer* **3**, 75–89 (2022). 35121990 10.1038/s43018-021-00299-1

[18] Yang, T. et al. Enolase 1 regulates stem cell-like properties in gastric cancer cells by stimulating glycolysis. *Cell Death Dis.* **11**, 870 (2020). 33067426 10.1038/s41419-020-03087-4 PMC7567818

[19] He, Y. et al. Targeting PI3K/Akt signal transduction for cancer therapy. *Signal Transduct. Target Ther.* **6**, 425 (2021). 34916492 10.1038/s41392-021-00828-5 PMC8677728

[20] Hoxhaj, G. & Manning, B. D. The PI3K-AKT network at the interface of oncogenic signaling and cancer metabolism. *Nat. Rev. Cancer* **20**, 74–88 (2020). 31686003 10.1038/s41568-019-0216-7 PMC7314312

[21] Tufail, M., Wan, W. D., Jiang, C. & Li, N. Targeting PI3K/AKT/mTOR signaling to overcome drug resistance in cancer. *Chem. Biol. Interact.* **396**, 111055 (2024). 38763348 10.1016/j.cbi.2024.111055

[22] Hao, C., Wei, Y., Meng, W., Zhang, J. & Yang, X. PI3K/AKT/mTOR inhibitors for hormone receptor-positive advanced breast cancer. *Cancer Treat. Rev.* **132**, 102861 (2025). 39662202 10.1016/j.ctrv.2024.102861

[23] Elstrom, R. L. et al. Akt stimulates aerobic glycolysis in cancer cells. *Cancer Res.* **64**, 3892–3899 (2004). 15172999 10.1158/0008-5472.CAN-03-2904

[24] Sun, Y. et al. Integrative plasma and fecal metabolomics identify functional metabolites in adenoma-colorectal cancer progression and as early diagnostic biomarkers. *Cancer Cell* **42**, 1386–1400.e1388 (2024). 39137727 10.1016/j.ccell.2024.07.005

[25] Shu, X. et al. Alpha-enolase (ENO1), identified as an antigen to monoclonal antibody 12C7, promotes the self-renewal and malignant phenotype of lung cancer stem cells by AMPK/mTOR pathway. *Stem Cell Res. Ther.* **12**, 119 (2021). 33579362 10.1186/s13287-021-02160-9 PMC7881626

[26] Xu, J., Ji, J. & Yan, X. H. Cross-talk between AMPK and mTOR in regulating energy balance. *Crit. Rev. Food Sci. Nutr.* **52**, 373–381 (2012). 22369257 10.1080/10408398.2010.500245

[27] Zhang, H., Steed, A., Co, M. & Chen, X. Cancer stem cells, epithelial–mesenchymal transition, ATP and their roles in drug resistance in cancer. *Cancer Drug Resist.* **4**, 684–709 (2021). 34322664 10.20517/cdr.2021.32 PMC8315560

[28] Laussel, C. & Leon, S. Cellular toxicity of the metabolic inhibitor 2-deoxyglucose and associated resistance mechanisms. *Biochem. Pharm.* **182**, 114213 (2020). 32890467 10.1016/j.bcp.2020.114213

[29] Chen, M. L. et al. A novel enolase-1 antibody targets multiple interacting players in the tumor microenvironment of advanced prostate cancer. *Mol. Cancer Ther.* **21**, 1337–1347 (2022). 35700013 10.1158/1535-7163.MCT-21-0285 PMC9662882

[30] Cappello, P. et al. Vaccination with ENO1 DNA prolongs survival of genetically engineered mice with pancreatic cancer. *Gastroenterology* **144**, 1098–1106 (2013). 23333712 10.1053/j.gastro.2013.01.020

[31] Zang, R. et al. Enhancement of diagnostic performance in lung cancers by combining CEA and CA125 with autoantibodies detection. *Oncoimmunology* **8**, e1625689 (2019). 31646071 10.1080/2162402X.2019.1625689 PMC6791432

[32] Song, Y. et al. Alpha-enolase as a potential cancer prognostic marker promotes cell growth, migration, and invasion in glioma. *Mol. Cancer* **13**, 65 (2014). 24650096 10.1186/1476-4598-13-65 PMC3994408

[33] Zhao, H. et al. Upregulation of glycolysis promotes the stemness and EMT phenotypes in gemcitabine-resistant pancreatic cancer cells. *J. Cell Mol. Med.* **21**, 2055–2067 (2017). 28244691 10.1111/jcmm.13126 PMC5571518

[34] Shen, Y. A., Wang, C. Y., Hsieh, Y. T., Chen, Y. J. & Wei, Y. H. Metabolic reprogramming orchestrates cancer stem cell properties in nasopharyngeal carcinoma. *Cell Cycle* **14**, 86–98 (2015). 25483072 10.4161/15384101.2014.974419 PMC4352969

[35] Ganapathy-Kanniappan, S. & Geschwind, J. F. Tumor glycolysis as a target for cancer therapy: progress and prospects. *Mol. Cancer* **12**, 152 (2013). 24298908 10.1186/1476-4598-12-152 PMC4223729

[36] Ke, R., Xu, Q., Li, C., Luo, L. & Huang, D. Mechanisms of AMPK in the maintenance of ATP balance during energy metabolism. *Cell Biol. Int.* **42**, 384–392 (2018). 29205673 10.1002/cbin.10915

[37] Tarrado-Castellarnau, M., de Atauri, P. & Cascante, M. Oncogenic regulation of tumor metabolic reprogramming. *Oncotarget* **7**, 62726–62753 (2016). 28040803 10.18632/oncotarget.10911 PMC5308762

[38] Qian, X. et al. Enolase 1 stimulates glycolysis to promote chemoresistance in gastric cancer. *Oncotarget* **8**, 47691–47708 (2017). 28548950 10.18632/oncotarget.17868 PMC5564598

[39] Sun, L. et al. Alpha-enolase promotes gastric cancer cell proliferation and metastasis by regulating AKT signaling pathway. *Eur. J. Pharm.* **845**, 8–15 (2019). 10.1016/j.ejphar.2018.12.035 30582908

[40] Sementino, E., Hassan, D., Bellacosa, A. & Testa, J. R. AKT and the hallmarks of cancer. *Cancer Res.* **84**, 4126–4139 (2024). 39437156 10.1158/0008-5472.CAN-24-1846

[41] Hosios, A. M. & Manning, B. D. Cancer signaling drives cancer metabolism: AKT and the Warburg effect. *Cancer Res.* **81**, 4896–4898 (2021). 34598998 10.1158/0008-5472.CAN-21-2647

[42] Xu, K. et al. Glycolysis fuels phosphoinositide 3-kinase signaling to bolster T-cell immunity. *Science* **371**, 405–410 (2021). 33479154 10.1126/science.abb2683 PMC8380312

[43] Xu, K. et al. Glycolytic ATP fuels phosphoinositide 3-kinase signaling to support effector T helper 17 cell responses. *Immunity* **54**, 976–987.e977 (2021). 33979589 10.1016/j.immuni.2021.04.008 PMC8130647

[44] Hopkins, B. D. et al. Suppression of insulin feedback enhances the efficacy of PI3K inhibitors. *Nature* **560**, 499–503 (2018). 30051890 10.1038/s41586-018-0343-4 PMC6197057

[45] Brooks, G. A. The science and translation of lactate shuttle theory. *Cell Metab.* **27**, 757–785 (2018). 29617642 10.1016/j.cmet.2018.03.008

[46] Cai, X. et al. Lactate activates the mitochondrial electron transport chain independently of its metabolism. *Mol. Cell* **83**, 3904–3920.e3907 (2023). 37879334 10.1016/j.molcel.2023.09.034 PMC10752619

[47] Eniafe, J. & Jiang, S. The functional roles of TCA cycle metabolites in cancer. *Oncogene* **40**, 3351–3363 (2021). 33864000 10.1038/s41388-020-01639-8

[48] Chen, J. et al. Lactate and lactylation in cancer. *Signal Transduct. Target Ther.* **10**, 38 (2025). 39934144 10.1038/s41392-024-02082-x PMC11814237

[49] Chen, Y. et al. Metabolic regulation of homologous recombination repair by MRE11 lactylation. *Cell* **187**, 294–311.e221 (2024). 38128537 10.1016/j.cell.2023.11.022 PMC11725302

[50] Chen, H. et al. NBS1 lactylation is required for efficient DNA repair and chemotherapy resistance. *Nature* **631**, 663–669 (2024). 38961290 10.1038/s41586-024-07620-9 PMC11254748

[51] Li, H. et al. AARS1 and AARS2 sense L-lactate to regulate cGAS as global lysine lactyltransferases. *Nature* **634**, 1229–1237 (2024). 39322678 10.1038/s41586-024-07992-y

[52] Huang, C. et al. Lactate promotes resistance to glucose starvation via upregulation of Bcl-2 mediated by mTOR activation. *Oncol. Rep.* **33**, 875–884 (2015). 25484022 10.3892/or.2014.3655

[53] Weiss, J. M. The promise and peril of targeting cell metabolism for cancer therapy. *Cancer Immunol. Immunother.* **69**, 255–261 (2020). 31781842 10.1007/s00262-019-02432-7 PMC7004869

[54] Xu, Y., Xue, D., Bankhead, A. 3rd & Neamati, N. Why all the fuss about oxidative phosphorylation (OXPHOS)?. *J. Med. Chem.* **63**, 14276–14307 (2020). 33103432 10.1021/acs.jmedchem.0c01013 PMC9298160

