# Supplementary material for: Alpha-enolase influences ATP pool of cytoplasm and lactate homeostasis by regulating glycolysis in gastric cancer
Source: Signal Transduct Target Ther. 2025 Oct 31;10:356. doi: 10.1038/s41392-025-02451-0 (PMC12575808; doi:10.1038/s41392-025-02451-0)
Supplement: Supplementary file 1 — Supplementary_Materials [file 41392_2025_2451_MOESM1_ESM.docx]

Supplementary Materials for

Alpha-enolase influences ATP pool of cytoplasm and lactate homeostasis by regulating glycolysis in gastric cancer

Xiong Shu, Shiya Liu, Ting Yang, Xuanyu Zhou, Gaigai Shen, Lixin Sun, Long Yu, Yuanting Cao, Yuliang Ran

Correspondence to: ranyuliang@cicams.ac.cn

**This PDF file includes:**

Figures. S1 to S10


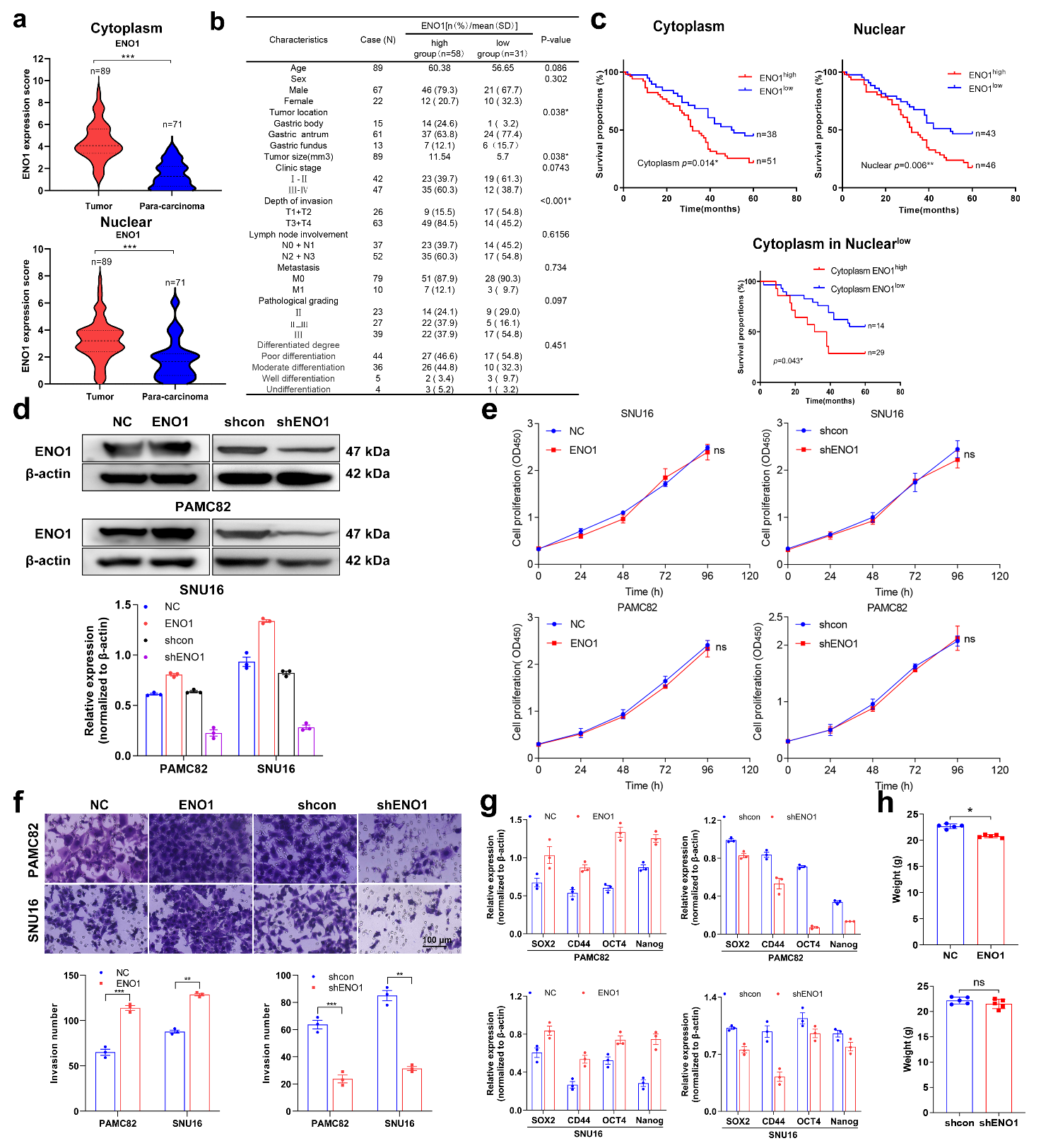


**Figure. S1. Highly expressed α-enolase (ENO1) related to poor patient prognosis and stem-like traits of gastric cancer (GC)**

(a) ENO1 expression between normal tissues and GC tissues in the cytoplasm and nuclear samples. (b) Clinical baseline characteristics of 89 gastric cancer patients stratified by ENO1 expression. (c) Analysis of the relevance of ENO1 protein expression with the survival rate of GC patients. High and low ENO1 expression levels in different sites were defined based on IHC immunoreactive score (IRS) analysis. The optimal cutoff value (IRS = 3.44) was determined using Youden’s J statistic, with samples classified as ENO1high (IRS ≥ 3.44) or ENO1low (IRS < 3.44) accordingly. (d) Protein expression of ENO1 in the PAMC82 and SUN16 GC cells was analyzed by western blotting. (e) Proliferation capacity in PAMC82 and SNU16 cells with stable ENO1 expression and silenced ENO1 expression. (f) Analysis of the invasion abilities of PAMC82 and SNU16 cells stably expressing ENO1 and shENO1, compared to normal control (NC) or shcon. Scale bar, 100 μm. (g) Quantitative analysis for expression of stemness markers analyzed by western blotting in PAMC82 and SNU16 cells stably expressing ENO1 and shENO1. (h) Lung weight in the lung metastasis model of SNU16 cells stably expressing ENO1 and shENO1, compared to normal control (NC) or shcon. Data were represented as the means ± standard error of the mean (S.E.M) of three independent experiments. *p < 0.05, ** p < 0.01, ns not significance.


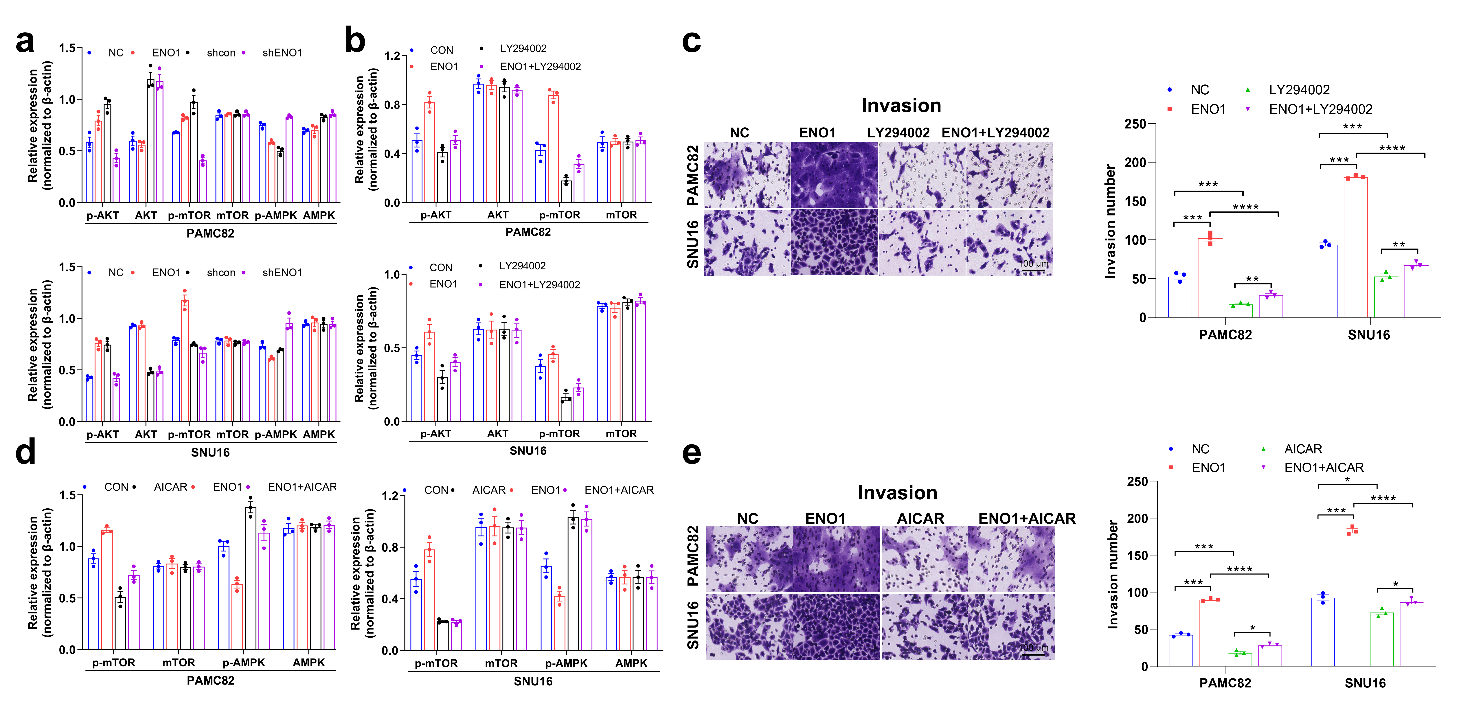


**Figure. S2. α-enolase (ENO1) promoted stem cell-like properties by mediating the PI3K/AKT and AMPK/mTOR pathways**

(a) Quantitative analysis for expressions of proteins-related PI3K/AKT and AMPK/mTOR pathways analyzed by western blotting in PAMC82 and SNU16 cells stably expressing ENO1 and shENO1. (b) Quantitative analysis for expressions of proteins-related PI3K/AKT pathway analyzed by western blotting. (c) Analysis of the invasion abilities in PAMC82 and SNU16 cells treated with PI3K inhibitor LY294002 (10 μM, 24 h). (d) Quantitative analysis for expressions proteins-related AMPK pathway analyzed by western blotting. (e) Analysis of the invasion abilities in PAMC82 and SNU16 cells treated with AMPK activator AICAR (0.5 mM, 24 h). Scale bar, 100 μm. Data are presented as mean ± SEM of three independent experiments. *p < 0.05, ** p < 0.01, *** p < 0.001, ****p < 0.0001.


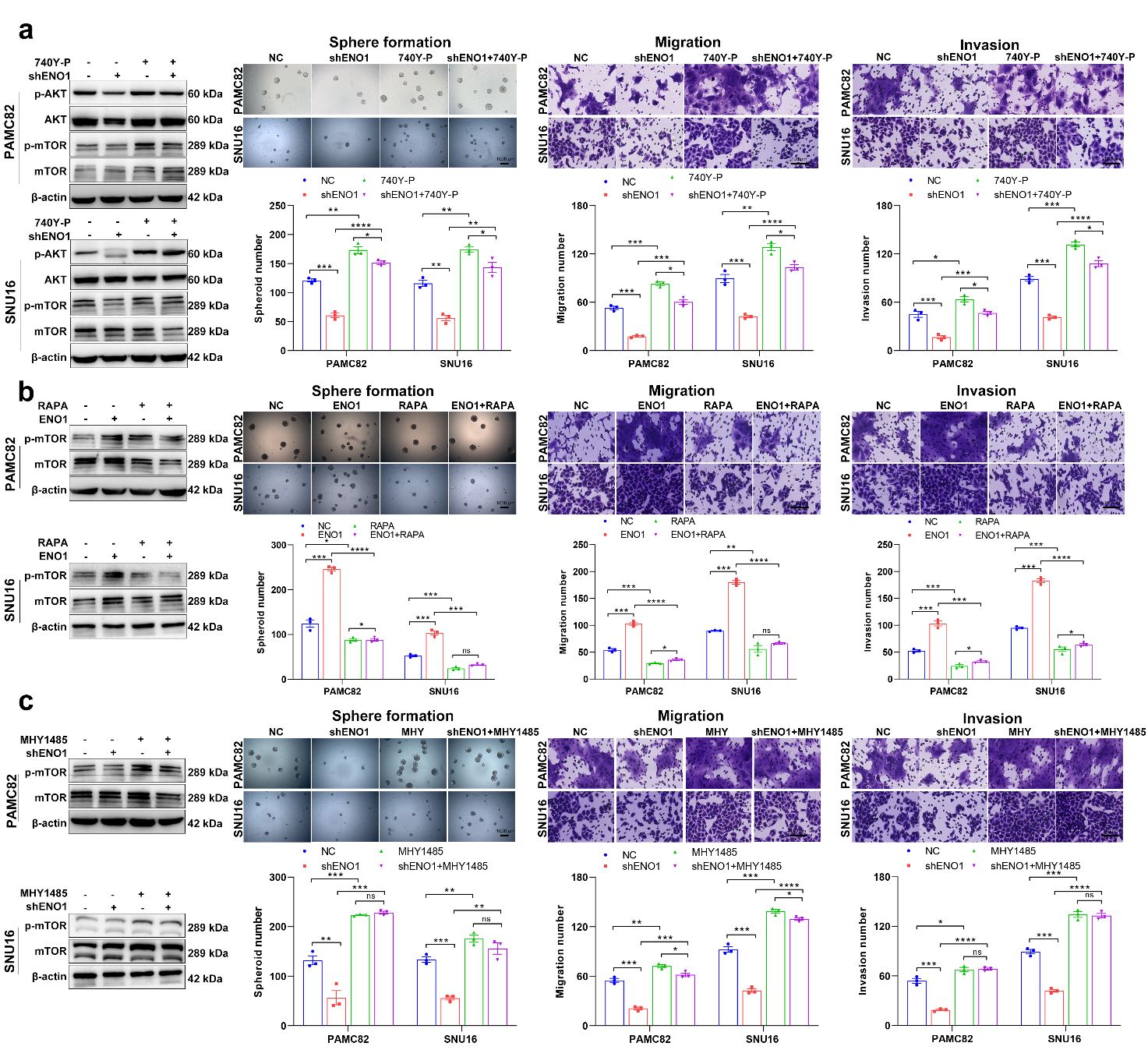


**Figure. S3. α-enolase (ENO1) related to the promotion of stem cell-like properties by mediating the PI3K/AKT and AMPK/mTOR pathways**

(a) Expression of proteins-related PI3K/AKT pathway was analyzed by western blotting, and analysis of the sphere-formation, migration, and invasion abilities in PAMC82 and SNU16 cells treated with PI3K activator 740Y-P (10 μM, 24 h). (b) Expression of proteins-related PI3K/AKT pathway was analyzed by western blotting, and analysis of the sphere-formation, migration, and invasion abilities in PAMC82 and SNU16 cells treated with mTOR inhibitor rapamycin (RAPA; 10 μM, 24 h). (c) Expression of proteins-related PI3K/AKT pathway was analyzed by western blotting, and analysis of the sphere-formation, migration, and invasion abilities in PAMC82 and SNU16 cells treated with mTOR activator MHY1485(10 μM, 24 h). Scale bar for sphere-formation, 1000 μm. Scale bar for migration/invasion, 100 μm. Data were represented as the means ± standard error of the mean (S.E.M) of three independent experiments. *p < 0.05, ** p < 0.01, *** p < 0.001, ****p < 0.0001, ns, no significance.


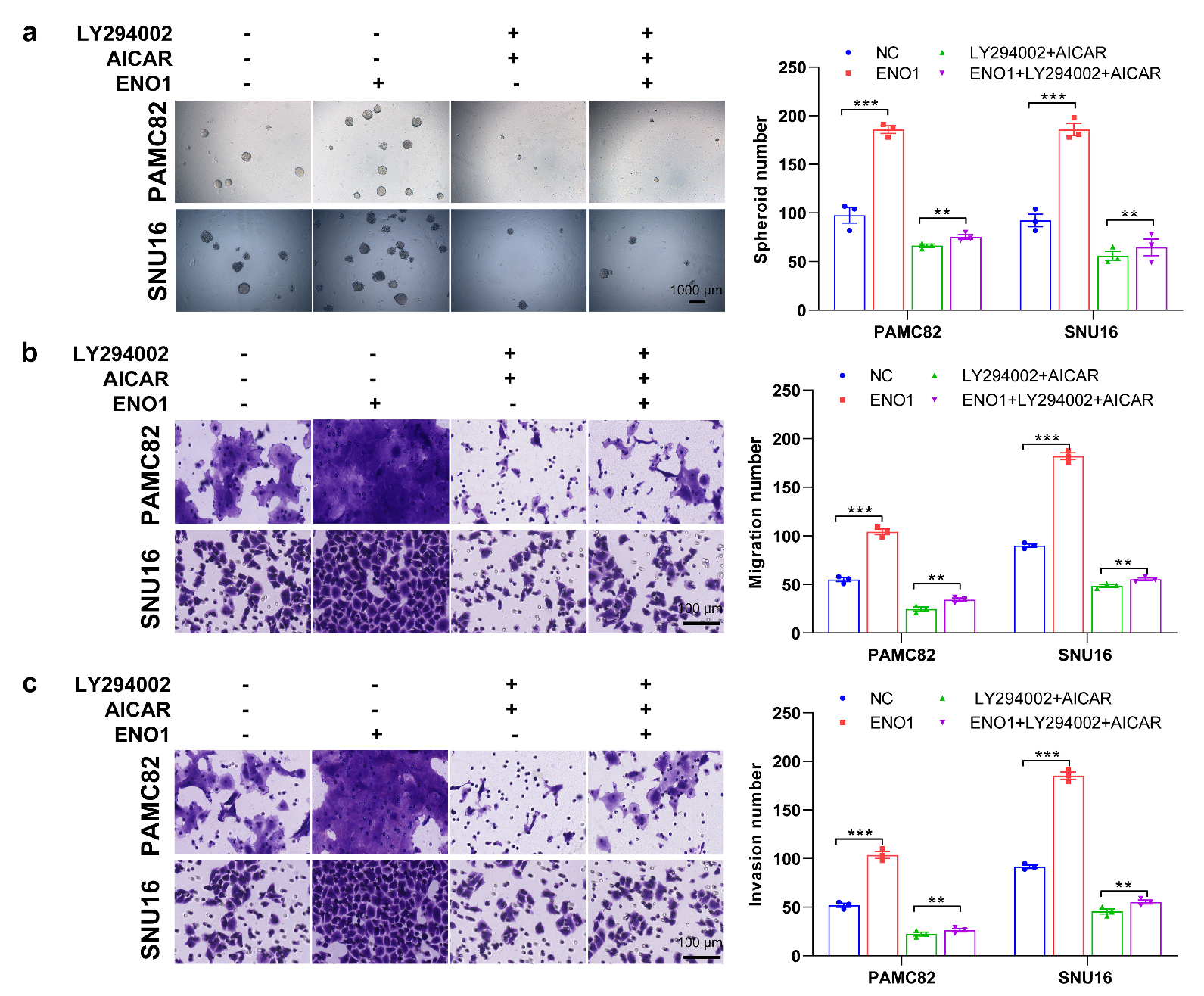


**Figure. S4. The synergistic effects of simultaneously activating the AMPK/mTOR and inhibiting the PI3K/AKT pathways on the biological behavior *in vitro***

The synergistic inhibition on sphere formation (a), migration (b), and invasion (c) abilities in PAMC82 and SNU16 cells using PI3K inhibitor LY294002 (10 μM, 24 h) and AMPK activator AICAR (0.5 mM, 24 h). Scale bar for sphere-formation, 1000 μm. Scale bar for migration/invasion, 100 μm. Data were represented as the means ± standard error of the mean (S.E.M) of three independent experiments. ** p < 0.01, *** p < 0.001.


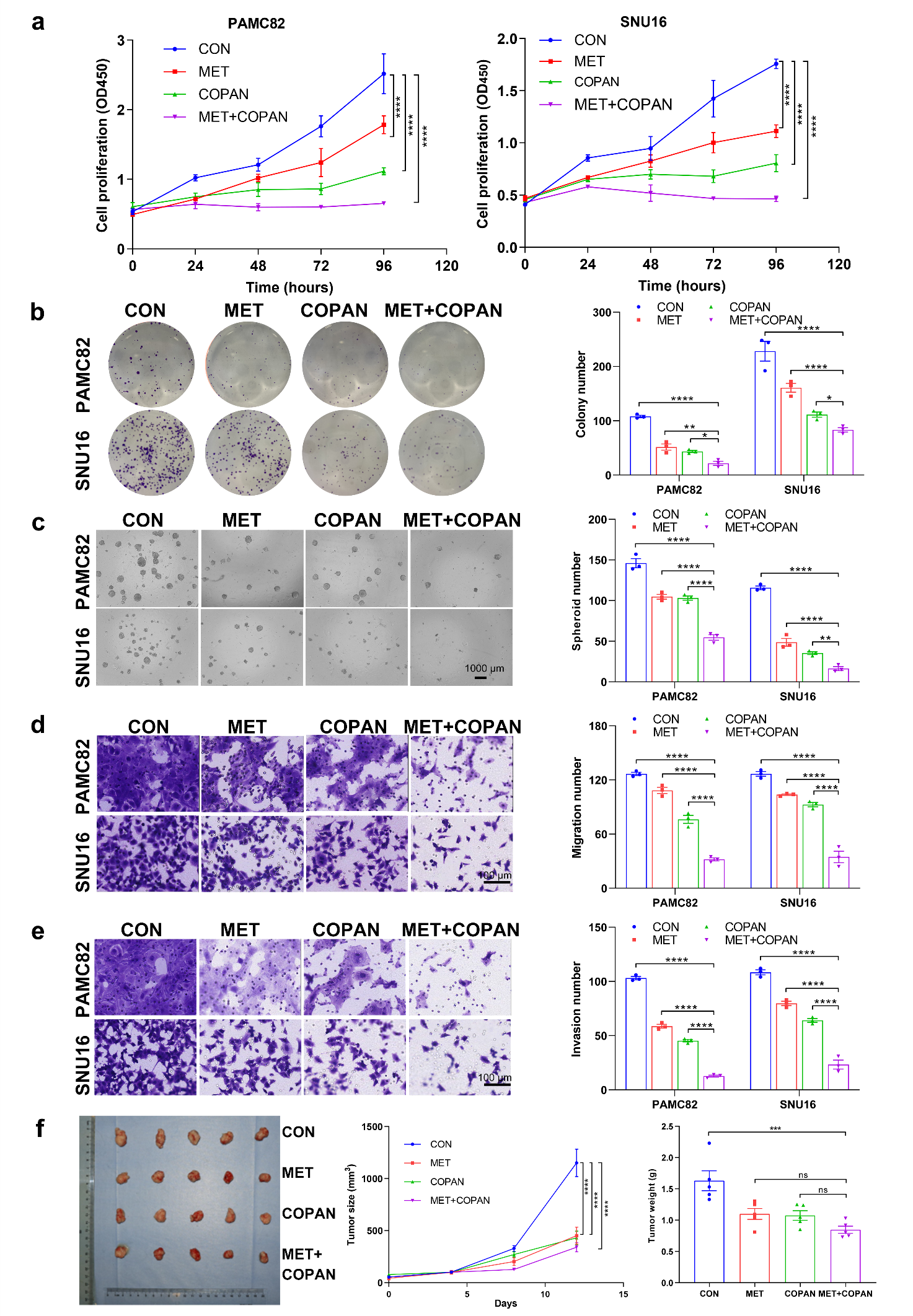


**Figure. S5. The combined therapeutic effects of simultaneously targeting the AMPK/mTOR and PI3K/AKT pathways *in vivo* and *in vitro***

The synergistic inhibition on cell proliferation (a), colony formation (b), sphere formation (c), migration (d), and invasion (e) abilities in PAMC82 and SNU16 cells using AMPK activating drug metformin (MET; 10 mM, 48 h) and PI3K inhibitory drug copanlisib (COPAN; 100 nM, 48 h). Scale bar for sphere-formation, 1000 μm. Scale bar for migration/invasion, 100 μm. (f) Representative macroscopic tumor images upon necropsy of mice with postimplant gastric cancer cells and posttreatment. Tumor volumes and weights were measured at the indicated time points in the tumor-implanted mice after treatment with metformin (350 mg/kg, intraperitoneal injection [i.p.]) and copanlisib (15 mg/kg, i.p.) twice weekly for 2 weeks. Data were represented as the means ± standard error of the mean (S.E.M) of three independent experiments. *p < 0.05, ** p < 0.01, *** p < 0.001, ****p < 0.0001, ns, no significance.


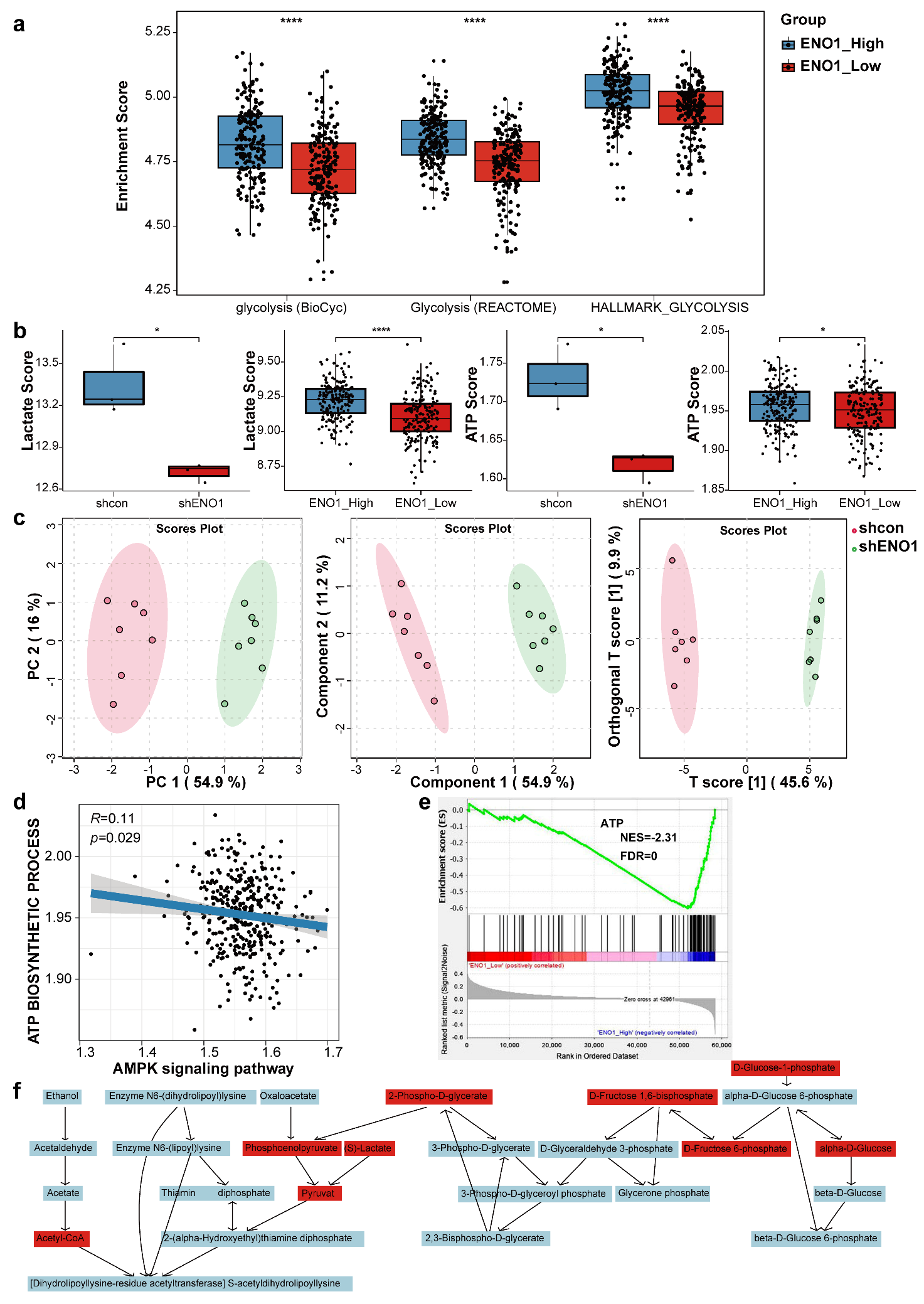


**Figure. S6. Bioinformatics analysis of the association between ENO1 expression and glycolysis**

(a) TCGA data showing a significant correlation between α-enolase (ENO1) and glycolysis enrichment score. (b) Analysis of lactate metabolism scores and ATP biosynthesis scores in shcon and shENO1 groups using RNA-seq and TCGA data. (c) PCA, PLS-DA, and OPLS-DA results of energy metabolism profiles in shcon and shENO1 group. (d) Scatter plots showing the correlation between ATP and AMPK signaling. The blue line represents the linear interpolation curve. The correlation coefficient R-value between the two was computed using Pearson’s coefficient correlation. (e) Gene set enrichment analysis (GSEA) plot showing ENO1 expression in association with the ATP. (f) Relevant metabolites in the glycolysis and gluconeogenesis pathways, with differential metabolites highlighted in red. *p < 0.05, **** p < 0.0001.


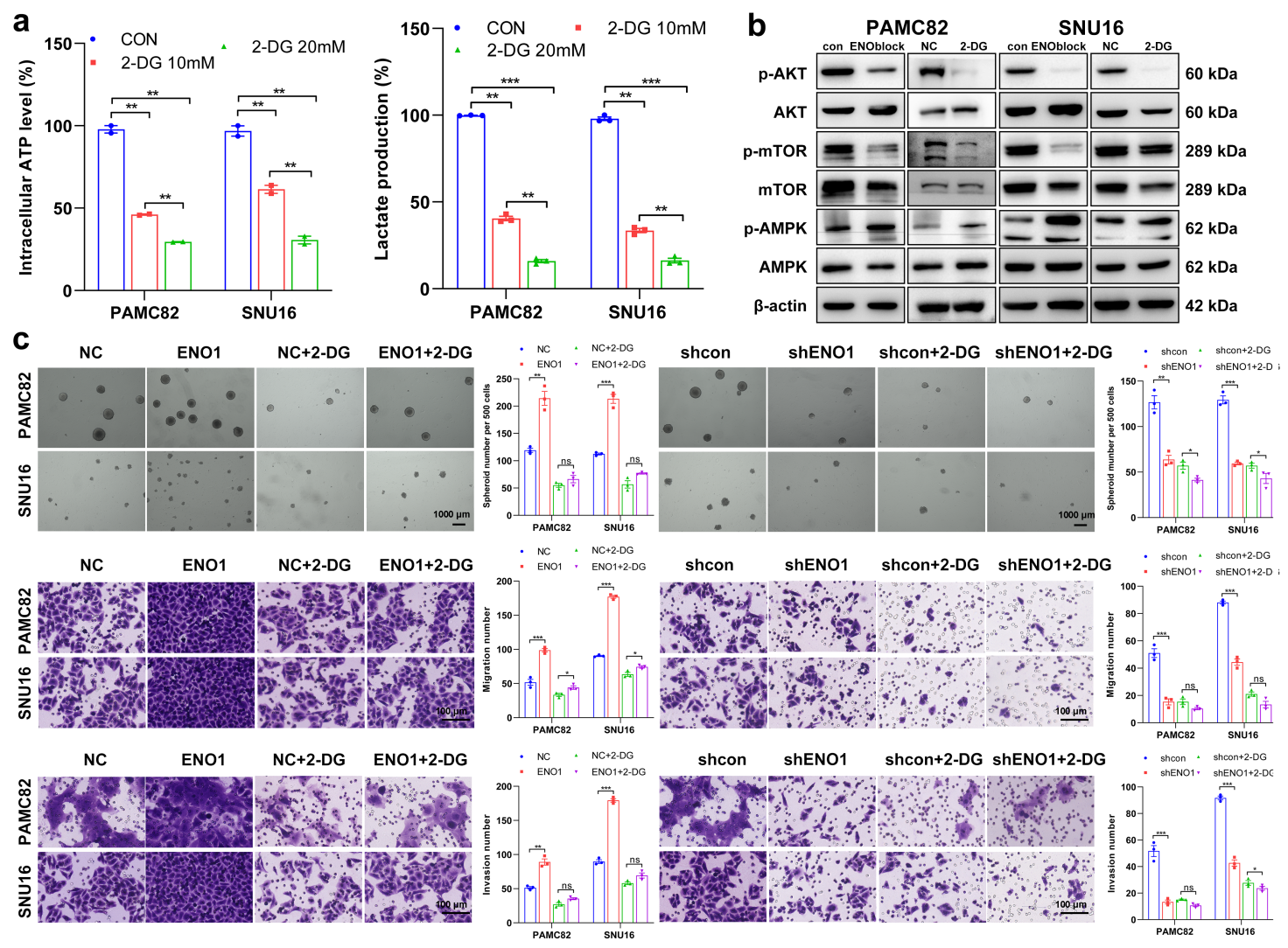


**Figure. S7. ENO1 promotes tumor stemness and metastasis depending on glycolysis**

(a) The lactate and intracellular ATP productions were measured in PAMC82 and SNU16 cells treated with glycolytic inhibitor 2-DG. (b) The expressions of proteins related to the PI3K/AKT and AMPK/mTOR pathways were monitored by western blotting in PAMC82 and SNU16 cells treated with glycolytic inhibitor 2-DG. (c) The effects of 2-DG on sphere formation, migration, and invasion ability in PAMC82 and SNU16 cells stably expressing ENO1 and shENO1. Scale bar for sphere-formation, 1000 μm. Scale bar for migration/invasion, 100 μm. Data were represented as the means ± standard error of the mean (S.E.M) of three independent experiments. *p < 0.05, ** p < 0.01, *** p < 0.001, ns, no significance.


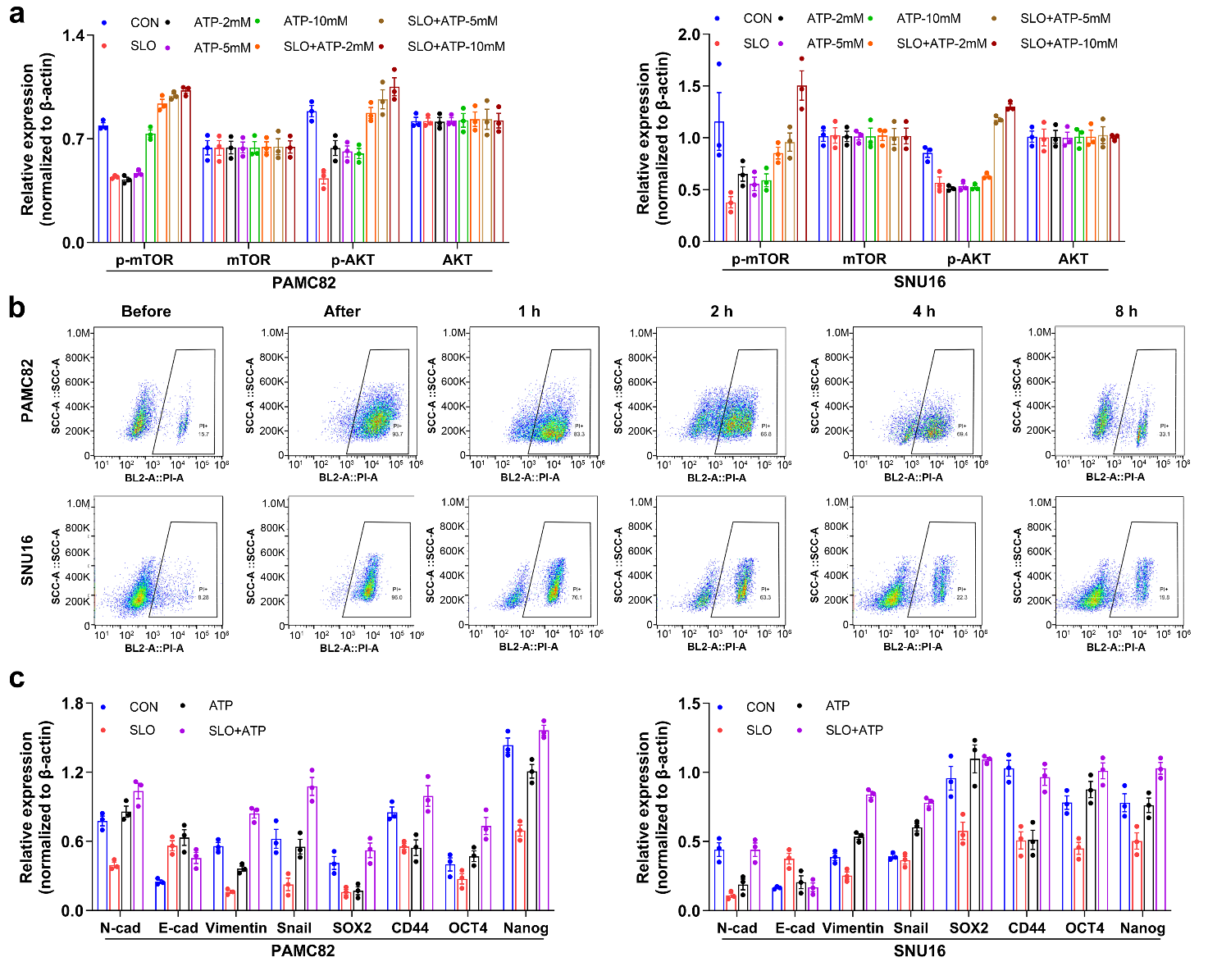


**Figure. S8. ENO1 promotes tumor stemness and metastasis depending on glycolysis**

(a) Quantitative analysis for protein expression related to PI3K/AKT signaling activation at different intracellular ATP concentrations post-permeabilization. (b) Flow cytometry analysis of membrane permeability recovery over time after SLO treatment. (c) Quantitative analysis for stemness and EMT marker expression after ATP addition and membrane recovery.


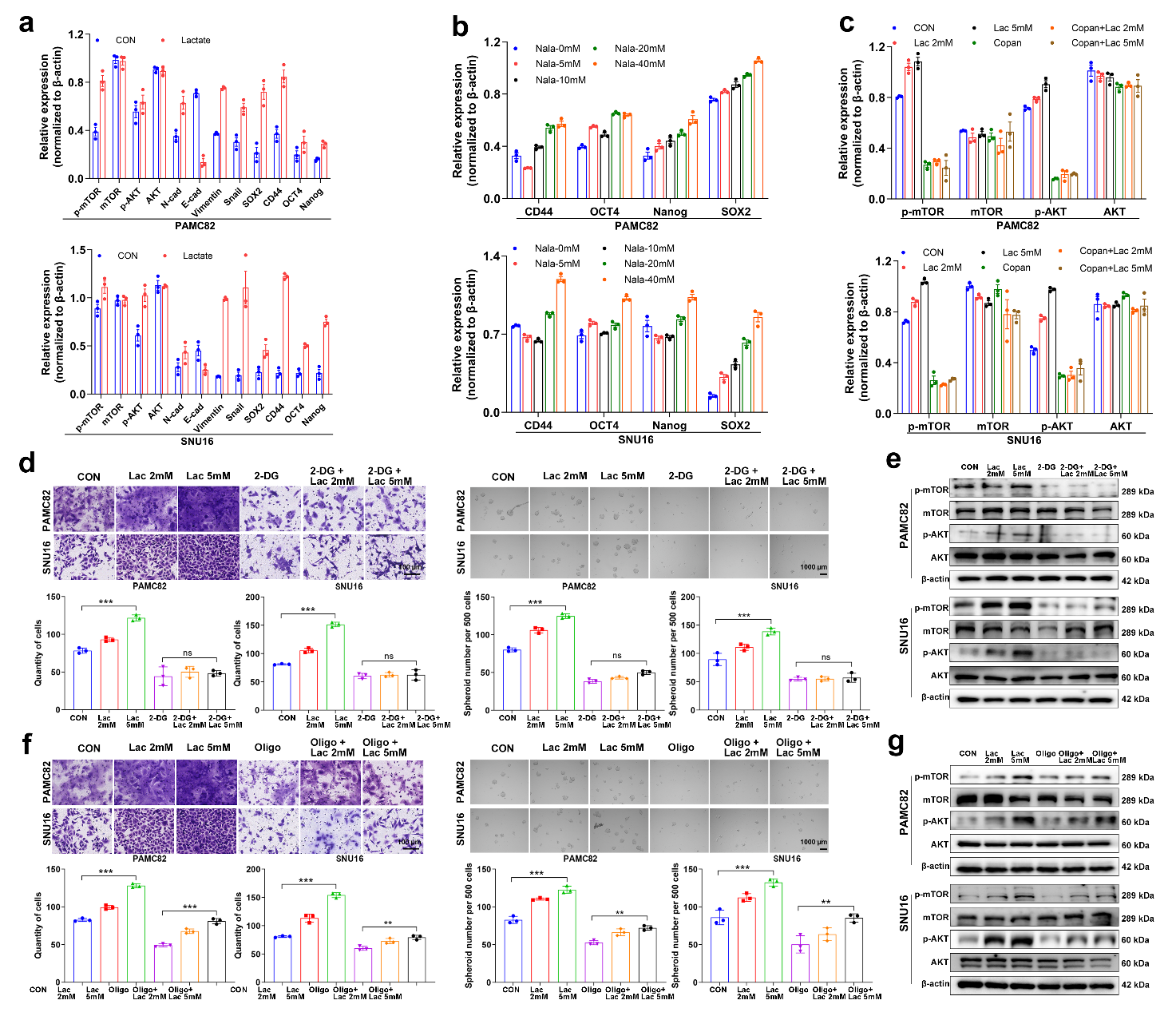


**Figure. S9. Lactate promotes tumor invasion and stemness in a dose-dependent manner, mediated by a glycolysis-derived ATP-dependent PI3K/AKT activation**

(a) Quantitative analysis for stemness and epithelial-mesenchymal transition (EMT)-related protein expression after lactate treatment. (b) Quantitative analysis for lactylation levels and stemness-related proteins after treatment with different sodium lactate concentrations. (c) Quantitative analysis for PI3K/AKT pathway-related protein expression. (d) Cell migration, sphere-forming ability, and PI3K/AKT pathway-related protein expression (e) under lactate treatment with or without the glycolysis inhibitor 2-DG. (f) Cell migration, sphere-forming ability, and PI3K/AKT pathway-related protein expression (g) under lactate treatment with or without the mitochondrial ATP production inhibitor oligomycin A. Scale bar for sphere-formation, 1000 μm. Scale bar for migration, 100 μm. Data are presented as mean ± SEM of three independent experiments. ** p < 0.01, *** p < 0.001, ns not significance.


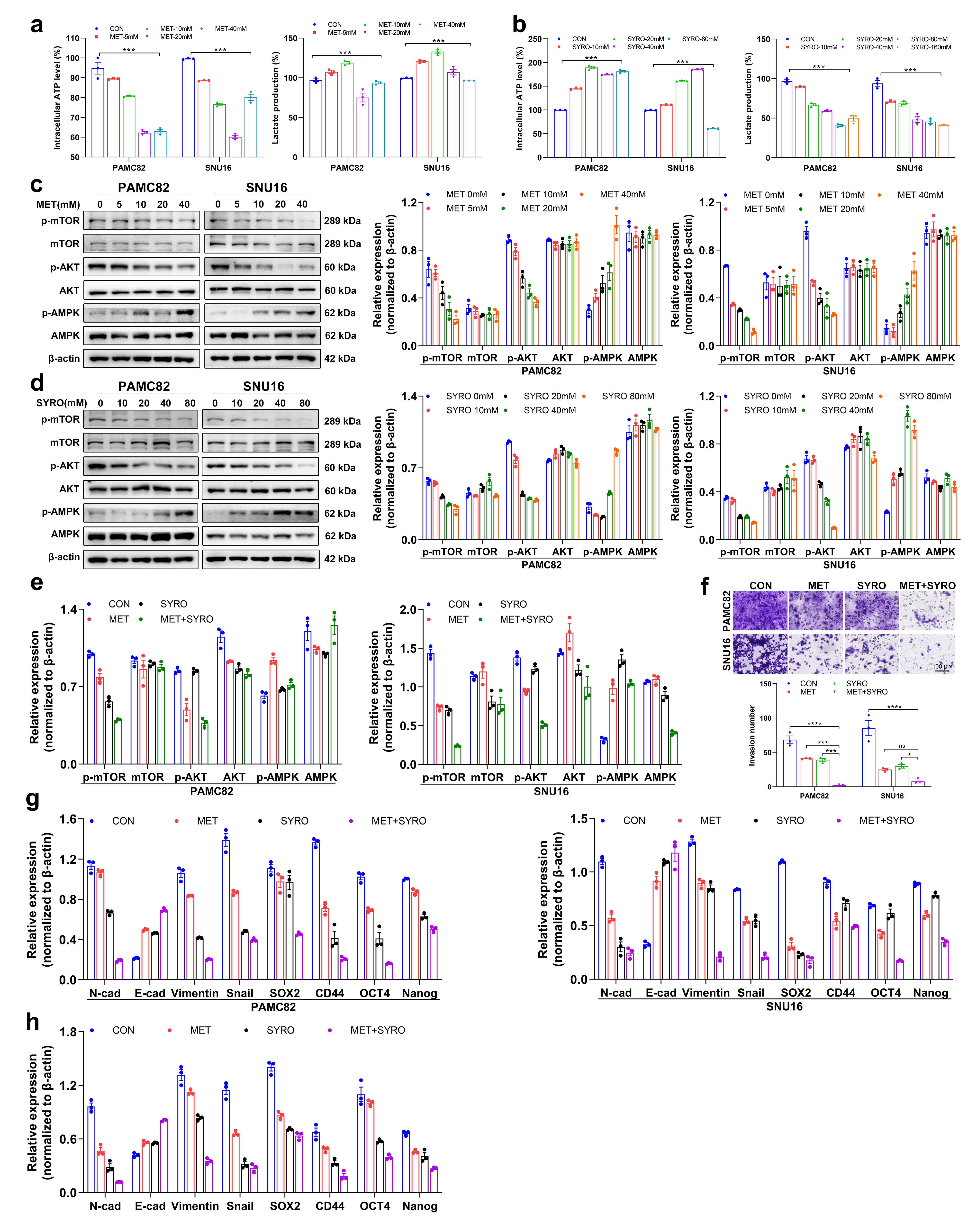


**Figure. S10. Simultaneous targeting of ATP and lactate inhibited the stemness in gastric cancer (GC) cells and tumor growth**

(a) The production of ATP and lactate was detected in PAMC82 and SNU16 treated with different concentrations of metformin (MET; ATP inhibitor). (b) The production of ATP and lactate was detected in PAMC82 and SNU16 treated with different concentrations of syrosingopine (SYRO; lactate transport inhibitor) after 48h. Expression of protein-related PI3K/AKT and AMPK/mTOR pathways was detected in PAMC82 and SNU16 treated with metformin (c) and syrosingopine (d) at different concentrations. (e) Quantitative analysis for protein expressions related to PI3K/AKT and AMPK/mTOR pathways tested in PAMC82 and SNU16 cells treated with metformin and/or syrosingopine. (f) The synergistic inhibition on invasion abilities in PAMC82 and SNU16 cells treated with metformin and/or syrosingopine. Scale bar, 100 μm. (g) Quantitative analysis for expression of stemness markers in PAMC82 and SNU16 cells treated with metformin and/or syrosingopine detected by western blotting. (h) Quantitative analysis for protein expressions of stemness markers tested using tumor samples collected from BALB/c nude mice treated with metformin (350 mg/kg, intraperitoneal [i.p.]) and/or syrosingopine (7.5 mg/kg, i.p.). Data were represented as the means ± standard error of the mean (S.E.M) of independent experiments. *p < 0.05, *** p < 0.001, **** p < 0.0001, ns not significance.
